# High Throughput Multispectral Image Processing with Applications in Food Science

**DOI:** 10.1371/journal.pone.0140122

**Published:** 2015-10-14

**Authors:** Panagiotis Tsakanikas, Dimitris Pavlidis, George-John Nychas

**Affiliations:** Laboratory of Microbiology and Biotechnology of Foods, Department of Food Science and Human Nutrition, School of Food, Biotechnology and Development, Agricultural University of Athens, Athens, Greece; Northwestern University Feinberg School of Medicine, UNITED STATES

## Abstract

Recently, machine vision is gaining attention in food science as well as in food industry concerning food quality assessment and monitoring. Into the framework of implementation of Process Analytical Technology (PAT) in the food industry, image processing can be used not only in estimation and even prediction of food quality but also in detection of adulteration. Towards these applications on food science, we present here a novel methodology for automated image analysis of several kinds of food products e.g. meat, vanilla crème and table olives, so as to increase objectivity, data reproducibility, low cost information extraction and faster quality assessment, without human intervention. Image processing’s outcome will be propagated to the downstream analysis. The developed multispectral image processing method is based on unsupervised machine learning approach (Gaussian Mixture Models) and a novel unsupervised scheme of spectral band selection for segmentation process optimization. Through the evaluation we prove its efficiency and robustness against the currently available semi-manual software, showing that the developed method is a high throughput approach appropriate for massive data extraction from food samples.

## Introduction

Food spoilage may be defined as the outcome of the biochemical activity of microbial processes, which will eventually dominate [1,2]. The factors that can influence the microbial activity and as a consequence, contribute in the food deterioration can be distinguished in two categories: (i) intrinsic (e.g. water activity, acidity, redox potential, available nutrients and natural antimicrobial substances) and (ii) extrinsic (storage conditions of temperature, humidity, atmosphere composition and packaging) [2,3]. Following this inherent complexity in food quality assessment, the acquisition of reliable information is a major challenge of the food industry, throughout the production, processing, and distribution chain [2,4–6]. Rapid non-destructive techniques for automatic monitoring of food processes in all stages of the food chain is a must, which is further enhanced by the fact that conventional microbiological analysis is time consuming, destructive and provides retrospective results. Recently, the potential of using non-destructive methods e.g. vibrational spectroscopy and surface chemistry sensors to overcome the limitations of conventional food microbiology as well as quality indices, gain more and more attention [7–10]. A promising new technique is the use of multi- and hyper-spectral imaging mainly because those sensors are able to give insight to the chemical composition of the samples. So, it is apparent that an efficient and robust image processing procedure is not only necessary but also a very crucial first step towards the unbiased and automated quality assessment and chemical composition of food samples.

The typical image processing techniques in the area of food research, include edge detection techniques, filters, trend removal, band ratio, thresholding techniques, digital morphology, texture, thinning and skeletonization algorithms. Image processing, in terms of informative area determination, is crucial in both spectral and image domain context since the extracted informative area is the basis of all the downstream analysis. The aforementioned methods perform pretty well when the goal is to detect defects on the surface of fruit/vegetables [11], but fail in the case of region of interest extraction, especially in complex types of food like meat, where the color exhibits severe variations. Finally, current methods have been developed either for images containing only the region of interest or they need manual processing. Additionally, they are case sensitive for every sample. Apart from the food industry and science there are numerous of works for multispectral image processing, mainly in the area of remote sensing [12]. Although the developed methodologies may be, in some degree, automated, their application on food sample applications is not straight-forward due to the use of different wavelengths, different definitions of regions of interest, and mainly due to the prior knowledge on the multispectral images considered, which is not the case in food samples (i.e. the different land areas of satellite images have specific features distinguishing them in each spectral band; an information known a priori).

Herein, we present a methodology for high throughput, automatic processing and information extraction from a multispectral imaging system, VideoMeterLab [13]. VideoMeterLab is a very common instrument for monitoring several properties of several kinds of food [14, 15]. Although it is accompanied with a software package for the acquisition and image analysis of the samples, VideoMeterLab’s software is not fully automated. So, it requires the user to label the different areas in order to extract the informative areas. This procedure, it is obvious that inherits the user’s subjectivity since the user is required to select the spectra (or spectra combination) that best contrasts the different areas and selects manually specific regions. So, one source of subjectivity is the choice of “optimal” image and a second is the labeling of the different areas. Additionally, in order to perform optimally, this procedure has to be repeated for every sample.

Considering the aforementioned limitations in terms of automated, high throughput and unbiased analysis on one hand and the lack of alternate software availability on the other, we developed a methodology to overcome this bottleneck and to enable high-throughput analysis of food samples. Another issue of complexity that concerns the food samples is that food exhibits severe variations that do not correspond necessarily to non-informative areas, this is the case in meat products, where dark areas are not always surrounding but may be meat area at very low oxygenation conditions. The method developed, is fully automated, robust, and reliable as we will show in the results section. In the corresponding processing system we embedded a phase of selecting the optimal channel combination without user intervention and more importantly, regardless the food sample. Then, another processing step, defines automatically and, individually for each input multispectral image, the appropriate threshold for the extraction of the informative sample areas, based on Gaussian Mixture Models [16] adapted to the application at hand.

## Materials and Methods

### Hardware and Data Introduction

**Fig 1:**
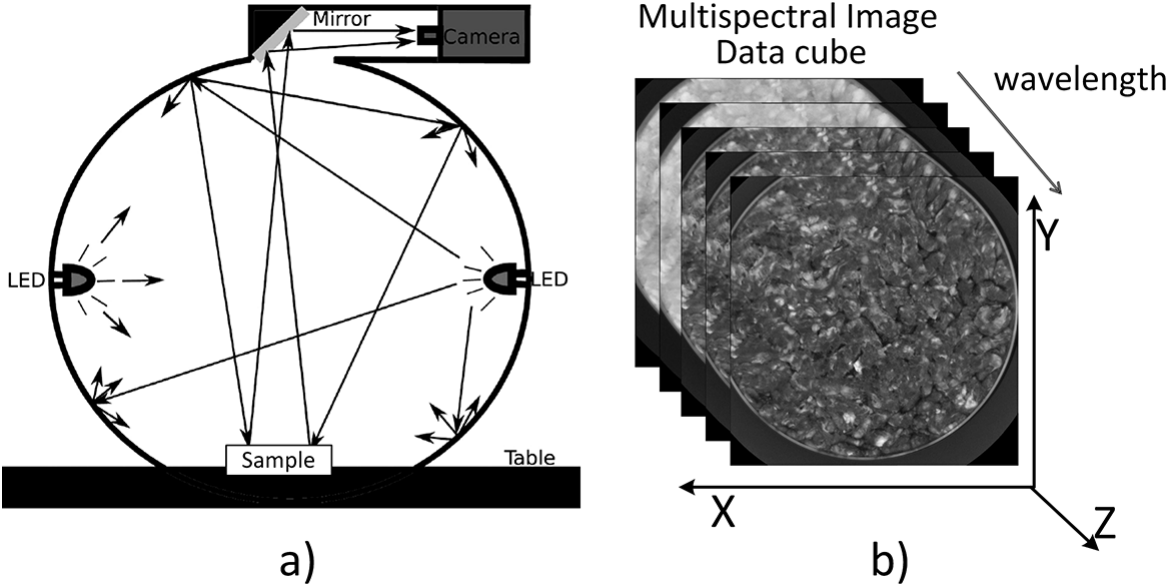
Instrument and Data Overview. (a) VideoMeterLab, (b) acquired data cube; here presenting a minced meat sample.

VideoMeterLab [17], captures multispectral images at 18 different wavelengths ranging from 405 to 970 nm. The acquisition system, records the surface reflections with a standard monochrome charged coupled device chip (CCD). A schematic illustration of the whole setup is shown in Fig 1(A). The sample is placed inside a sphere, called Ulbricht sphere, which has a matte white coating so as to ensure a diffused and spatially homogenous illumination of the sample. At the rim of the sphere, light emitting diodes (LEDs) with narrow-band spectral radiation distribution are positioned side by side. During data acquisition, the diodes are strobing successively, resulting in a monochrome image with 32-bit floating point precision for each wavelength. Finally, a data cube of spatial and spectral data for each sample of size *m x n x 18* (where *m x n* is the image size in pixels) is acquired. The whole system, is developed in order to guarantee the reproducibility of the collected images, and so it can be used in comparative studies of time series studies, or across a large variety of different samples. Summarizing, it is obvious that the acquisition system is well fitted and highly efficient for food quality assessment and monitoring through food process and distribution chain.

The acquisition process results to a plethora of data, either representing samples in a time series experiments or samples under different conditions and/or treatment (e.g. packaging conditions). Each sample image cube contains spatial as well as spectral information (please refer to Fig 1(B)). Spectral information for every pixel lies at the spectral axis, whereas spatial information (x-y plane) gives the reflectance value of the sample at the pixel’s location and at the specific wavelength. It is apparent that each multispectral image, besides the informative sample-specific area, includes irrelevant information, corresponding to the surrounding (e.g. petri dish, packaging material) and in the case of meat samples, to fat and connective tissue areas. Having this in mind, we can safely conclude that an automated and bias free method for extracting the informative areas from the data is more than a necessity, especially if someone wants to process a vast amount of data.

### Prior Approach and Limitations

As already stated, there are no software packages currently available for the segmentation of multispectral images acquired by VideoMeterLab, at least as far as we know. Nevertheless, there are methods for automated food sample processing from RGB images (e.g. [18]). Those methods, are not applicable to multispectral images, because RGB images use wide bands for each color (red, blue and green), while in the case of multispectral images the bands are much narrower (e.g. two or more bands may represent several sub-bands of the same color, e.g. red). VideoMeterLab is accompanied with software for image analysis and foreground detection, but does not enable automation and bias free analysis. The VideoMeterLab software (version 2.12.39), apart for controlling the acquisition operation of the instrument, includes several segmentation processes for the informative area extraction, ranging from simple thresholding to more sophisticated methods (as described next). The most efficient method maximizes the contrast between the sample material and irrelevant objects, in order to enable a threshold operation [9]. This by itself, is not adequate for the segmentation, so Canonical Discriminant Analysis (CDA) [19] is employed as a supervised transformation building method so as to divide the image into regions of interest [20]. This way a segmented image is produced, where the region of interest is used for the extraction of spectral data that will then be used for subsequent data analysis.

The aforementioned procedure has several shortcomings that mainly concerns the lack of automated functionality, even for repeated measurement experiments, i.e. experiments with the same sample and/or the same sample type. This lack of automation lies on the fact that the user has to label manually the different regions of the image, according to user’s subjective judgment on whether it is informative (sample area) or not (surrounding area–non informative areas). This labeling task is not trivial and subjective, since it depends greatly on the inherent artifacts on the various sample types. In the case of meat samples, we have the fat regions (different reflection for different kinds of fat), samples of different degrees of spoilage and/or oxidation can be easily misclassified as informative areas or not. In addition, at the labeling phase, the user has to label several different regions with the same, region specific label, so as the system can recognize all possible different “states” of the same category (either background or foreground). Finally, another major source of artifacts that can lead to unreliable segmentation is the food packaging that in most cases is “contaminated” with sample and sample byproducts. So, we can conclude that even using a supervised method, the existing segmentation module, needs very careful calibration for each sample, and still its functionality is not guaranteed, mainly due to human errors and biases.

### Automated Segmentation Method

**Fig 2:**
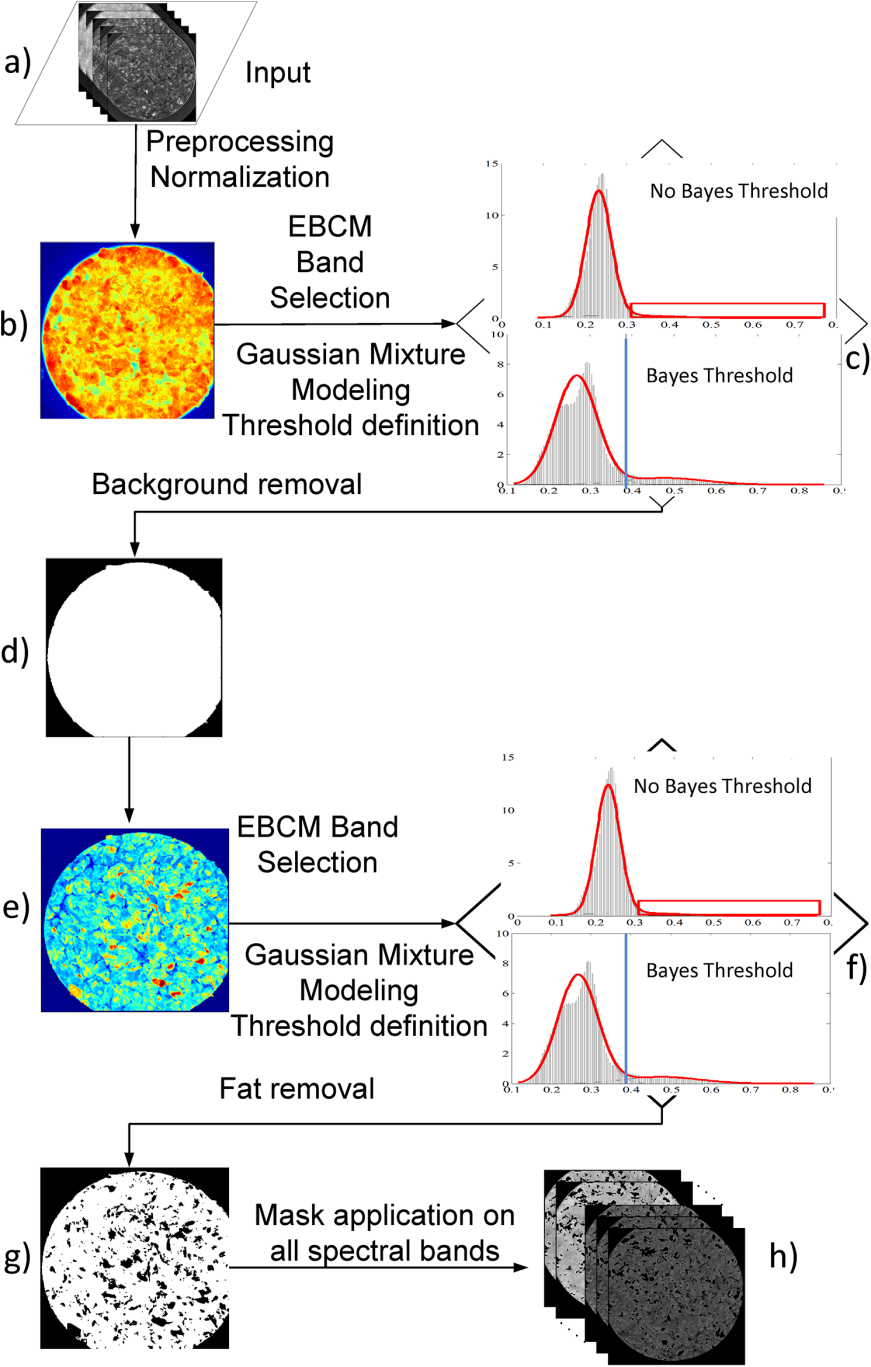
Overview of the automated segmentation. (a) Original image input, preprocessing and normalization, (b) EBMC based wavelength band selection, (c) mixture modeling for threshold definition for background removal, (d) binary mask of meat product sample, (e) EBMC based wavelength band selection for fat and connective tissue detection using (f) mixture modeling for threshold definition, (g) binary mask of final informative area and (h) application of the final binary mask to all 18 spectral bands.

Herein, we will present in detail the workflow of the automated analysis and region of interest extraction (as presented in Fig 2). The input is a multispectral image or a series of images of the same kind of food product (e.g. minced meat, fillet meat, etc.). A preprocessing step follows, where a two phase normalization of the input data takes place, one at the basis of an image at each wavelength (x-y plane, Fig 1(B)) and one at the basis of spectral axis (z axis, Fig 1(B)). The first phase concerns the normalization of each spectral image separately in the range [0,1] using Eq 1, in order to combat the large variation of the pixels’ values, in each spectral image. Often these values do not exploit the whole dynamic range of reflectance i.e. from 0% to 100% and Eq 1 transforms the data so as to exploit the whole dynamic range.

$$I_{w}^{'}\left(x,y\right)=\frac{I_{w}\left(x,y\right)-\text{min}\;\left(I_{w}\right)}{\text{max}\;\left(I_{w}\right)-\text{min}\;\left(I_{w}\right)}$$ (1)

where *I*_*w*_(*x*, *y*) is the value of the pixel *(x*,*y)* of the image *I*_*w*_ at wavelength *w*. The second pre-processing phase, Standard Normal Variate (SNV) normalization [21] (Eq 2), is used in order to enhance the data quality, reduce the correlated information across the different wavelengths and eliminate the inherent, due to the acquisition process, multiplicative noise.

$$I\_SNV\;\left(x,y\right)=\frac{I\;\left(x,y\right)-\text{mean}\;\left(I\right)}{\text{std}\;\left(I\right)}$$ (2)

where *I(x*,*y)* is the spectrum of pixel *(x*,*y)* of the multispectral image *I*.

Next, follows the discrimination and removal of the non-informative area (i.e. surrounding, Petri dish, sample byproducts lying on the packaging). Since we have 18 different wavelength images, where each one holds different information for the sample, a critical step is to recognize the wavelengths and combination of spectral images pair (*I*_*diff*_) that maximizes the contrast between the two areas, i.e. sample vs. non sample. At this point we must note that for each sample, different spectral images may exhibit the maximum contrast and thus the procedure developed, is data driven and thus data depended. The choice of the pair of spectral images is automatically determined by employing a contrast measure, EBCM used in [22]. In essence, the edge-based contrast measure (EBCM) outputs a large value for an image with a lot of edges and small values for the opposite:

$$EBCM\;\left(I_{diff}\right)=\sum_{x=1}^{M}\sum_{y=1}^{N}c\;\left(x,y\right)/MN$$ (3)

where *c(x*,*y)* is the contrast for a pixel of the image *I*_*diff*_, computed as the average value of the pixel’s *(x*,*y)* grey level weighted by their edge values, and *MxN* is the image size. So, *c(x*,*y)* is computed as:

$$c\left(x,y\right)=\frac{\left|I_{diff}\left(x,y\right)-e\left(x,y\right)\right|}{\left|I_{diff}\left(x,y\right)+e\left(x,y\right)\right|}$$ (4)

where the mean edge grey level *e(x*,*y)* is defined as:

$$e\left(x,y\right)=\frac{\sum_{\left(k,l\right)\in N\left(x,y\right)}g\left(k,l\right)I_{diff}\left(k,l\right)}{\sum_{\left(k,l\right)\in N\left(x,y\right)}g\left(k,l\right)}$$ (5)

where *N(k*,*l)* defines the neighborhood of the pixel *(x*,*y)* (a 3x3 neighborhood is used) and *g(k*,*l)* is the edge value of the *(k*,*l)* pixel after applying the Sobel operator [23] on the image *I*_*diff*_.

In [22], the authors used this in order to measure and evaluate the performance of different contrast enhancement methods. Here, we use it as a method for feature selection, i.e. spectral band selection, and thus we turn around the physical meaning of the EMBC value. In essence, we use the pair of spectral images that exhibits the minimum EBCM measure rather than the maximum. The rational for doing so, is that when we have the least edges, we have a somewhat “blurry” image, but the distinct regions are better distinguishable than in an image with much more edges that catch every little detail (maximizing its EBCM measure). Fig 2(B) and 2(E), show the pair combination of the selected spectral images. We can see that we get a clear separation of sample area from its surrounding in Fig 2(B) and a rather clear distinction between meat and fat areas in Fig 2(E). S1 File presents an example of the use and application of EBCM measure for spectral band selection.

At the core of our methodology, and in order to define an appropriate threshold, we employ a data driven approach, borrowed from machine learning, the Gaussian Mixture Modeling [16]. Mixture models allow for a model-based approach to unsupervised clustering. Intuitively, Gaussian mixtures can simply be seen as models able to represent arbitrarily complex multimodal probability density functions (pdfs). This makes them an ideal tool for representing complex class-conditional pdfs, also in supervised learning scenarios [24, 25]. Let ***Y*** *= [Y*_*1*_,*…*,*Y*_*d*_ *]* ^*T*^ be a random variable of dimensionality *d*, with ***y*** *= [y*_*1*_,*…*,*y*_*d*_ *]* ^*T*^ representing a specific realization of ***Y***. Then ***Y*** has a finite mixture distribution if its probability density function can be written as:

$$f_{Y}\left(y|\Theta \right)=\sum_{m=1}^{C}\pi_{m}f_{Y}\left(y|\theta_{m}\right)$$ (6)

where *f*_*Y*_ *(* ***y|Θ*** *)* is a component density function, *C* is the number of components and *π*_*m*_ are their mixing probabilities. Having a specific density function, common for all the mixture components, yields a parameter set ***Θ*** *= {θ*_*1*_,*…*,*θ*_*c*_, *π*_*1*_,*…*,*π*_*c-1*_ *}* where $\pi_{c}=1-\sum_{m-1}^{c-1}\pi_{m}$. For the developed methodology the Normal (Gaussian) density function was considered: *f*_*Y*_(***y***|*θ*_*m*_) = *N*(***y***|***μ***_*m*_, ***Σ***_*m*_), with a general covariance matrix ***Σ***_*m*_ and mean vector ***μ***_*m*_. So, in that case the component parameters are ***θ***_*m*_ *= (* ***μ***_*m*_,***Σ***_*m*_ *)*. The maximum likelihood (ML) estimate of the mixture parameters ***Θ***, based on a set of *n* independent observations ***y*** *= {y* ^*(1)*^,*…*, *y* ^*(n)*^ *}*, is defined as:

$$\hat{\Theta }={argmax}_{\Theta }L\left(\Theta ,y\right)$$ (7)

where *L*(**Θ**) is the likelihood of the dataset under the model. As it is known, the maximum likelihood estimate $\hat{\Theta }$ does not have, in general, a closed form expression but may be approximated iteratively by applying the Expectation Maximization (EM) algorithm [26].

GMMs have been modified so as to be used in an unsupervised manner [27], meaning that no training is required, and the final number of classes is defined automatically based on data distribution on the variable space. Intuitively, GMMs can identify the “natural” number of distribution sources (and their parameters) that potentially could have generated the observed data. In our methodology we employ the 1-dimensional GMMs since our observations are the monochromatic pixels’ intensities. The GMM is applied on the histogram where we expect to have at maximum, two distributions, one representing the informative area and one either representing the surrounding (Fig 2(C)) or the fat/connective tissue (Fig 2(F)). There is always the possibility that the surrounding area is much less than the foreground (e.g. in a fatless sample) and the number of those data cannot form a distinct separable population (i.e. a separate data distribution from the foreground). Thus, the algorithm should be able to automatically decide whether the distribution of the data points is explained better by one or by a mixture of two Gaussian distributions. This is achieved through the Minimum Message Length (MML) criterion for automated model selection [27]. So, if the algorithm decides that there are two different populations, then the threshold is determined automatically under the Bayes rule [28]. Otherwise, the kurtosis of the resulted Gaussian probability distribution function is calculated, and the threshold is set so as to reject the pixels with intensities belonging to the larger tail (see Fig 2(C) and 2(F)). At the last step, the resulted final mask (Fig 2(G)) is applied to all spectral bands and we get the final segmented multispectral image (Fig 2(H)).

### Datasets and Evaluation Procedure Description

#### Datasets

We used several images, acquired with VideoMeterLab, for different meat types and food products, so as to evaluate the efficiency and generality of the methodology for samples with varying quality and appearance. So, we used five different datasets; minced meat, beef fillet meat, pork fillet meat, vanilla crème and table olives (please refer to S2 File for representative sample images). In order to be even more unbiased and “chaotic”, as in real life, we also added intra-sample variance, i.e. the samples of each datasets are acquired under different conditions of temperature, packaging, storage time and degree of microbiological contamination. This way, we ensure that whatever segmentation result is not biased to the input, since for different conditions the samples are degraded differently and the reflections values follow this degradation. From the above, it is apparent that the evaluation scheme selected, is unbiased and the segmentation results should give a reliable measure of its efficiency.

For this study 147 meat samples from seven independent experimental procedures which mainly belong to 50 raw minced and 97 meat fillets were used: **(i)** 24 "sterile" pork fillets which were incubated at 4° and 10°C aerobically and under modified atmosphere packaging (80% O_2_:20% CO_2_) **(ii)** 23 "sterile" pork fillets inoculated with the specific spoilage microorganism *Pseudomonas putida* at 10^2^ log_10_CFU/cm^2^ inoculum, which were incubated at 4° and 10°C aerobically and under modified atmosphere packaging (80% O_2_:20% CO_2_) **(iii)** 10 sterile beef fillets including sample at time point 0 and the others that were incubated at 2°, 8° and 15°C, **(iv)** 21 naturally contaminated beef fillets, which were incubated at 2°, 8° and 15°C, **(v)** 19 naturally contaminated beef fillets inoculated with different inocula of *Salmonella* TYMPHIMURIUM, corresponding to 10^3^, 10^4^ and 10^5^ log_10_CFU/cm^2^ **(vi)** 30 minced meat samples (10 beef, 10 pork, 10 mixed in 70–30% of beef-pork), which were obtained from different retailers and had high fat percentage grinded together with meat muscle and **(vii)** 30 minced mix meat corresponding to different percentages (100%, 80%, 50% and 30% beef to pork ratio) of adulteration. In the last case, minced meat was derived by grinding separate beef and pork fillets manually, which were previously trimmed of extraneous connective tissue and fat by an expert butcher. This allowed all white objects to be taken as marbling fat. Steak trimming prevents intramuscular fat being mistaken for marbling fat. All meat samples were derived from *Longissimus* muscle of normal pH, obtained. In order to obtain sterile fillets, a large portion of *Longisimmus* muscle was used, sprayed with ethanol 100% and flamed. Under a laminar flow cabinet the external part was removed aseptically and the internal sterile was cut into smaller pieces and placed onto Petri dishes.

The datasets of vanilla crème consisted of 20 samples stored at 4°C for a period of time of one month, while the table olives dataset consisted of 20 samples stored at room temperature for a period of time of four months.

#### Evaluation Procedure Description

The evaluation procedure is based on as much as possible objective measures since the “ground truth” is unknown. So, an expert user used VideoMeterLab software to identify the informative areas of the samples (manual segmentation). From now on we will consider those results, as the “ground” truth. All the datasets were analyzed by the two methods, namely VideoMeterLab (VM) and the Automatic Segmentation (AS). The evaluation is based on several criteria (partially overlapping and not mutually excluded) that should give a safe conclusion on the comparison. Those criteria are: 1) informative area size, 2) mean values of spectral information of the informative areas for each wavelength, 3) standard deviation of the corresponding mean values, 3) colocalization percentage on the basis of VM results. Raw and processed images are available at: http://dx.doi.org/10.5281/zenodo.31442.

## Results and Discussion

**Fig 3:**
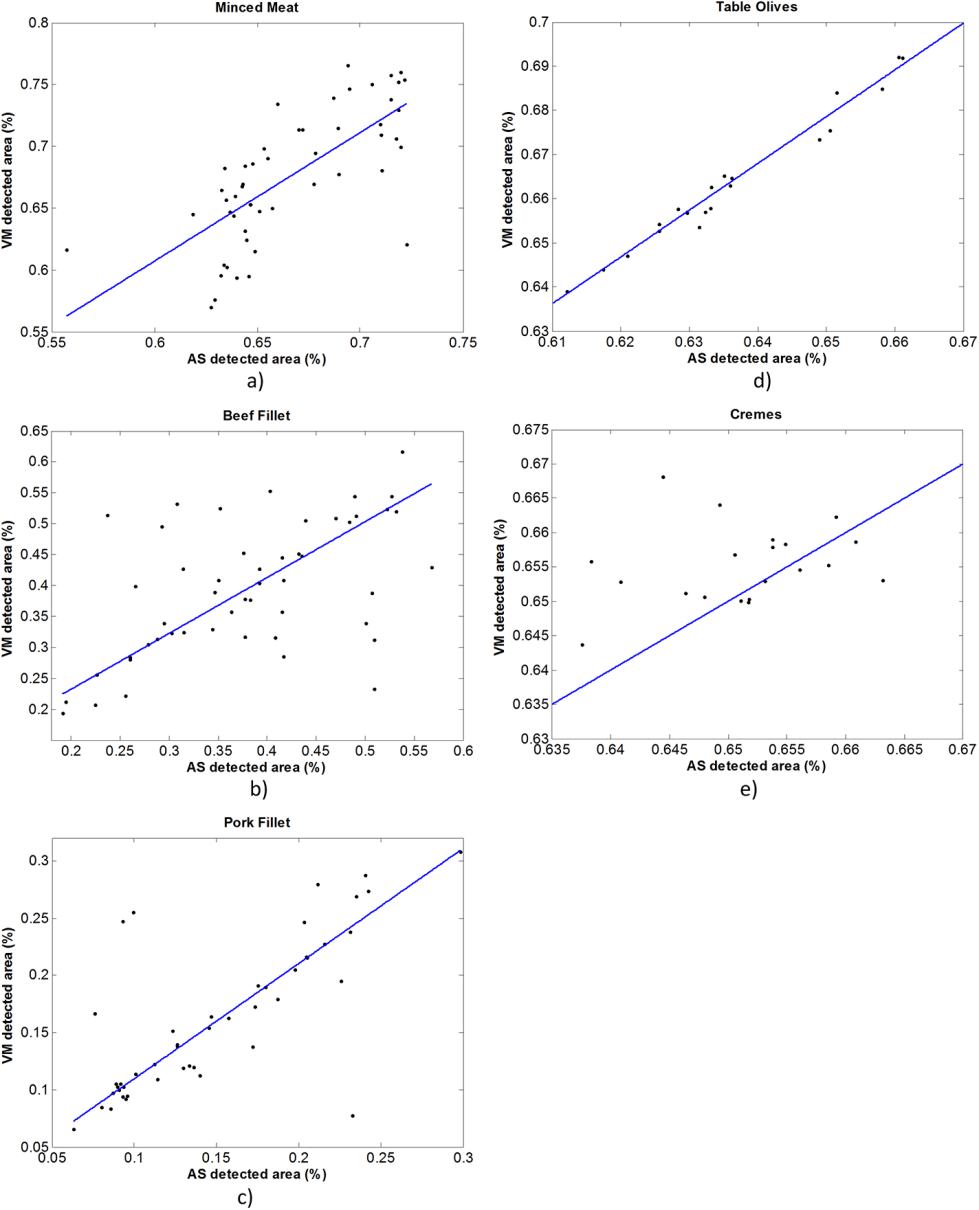
Regression Analysis. Linear regression of the area sizes (percentages, i.e. ratio of informative detected areas to the total area of the image) resulted by the two methods VM (VideoMeterLab) and AS (Automated Segmentation).

In this section, the results from the automated segmentation (AS) and VideoMeterLab software (VM) are presented. A first analysis is done based on the informative areas sizes (please refer to Fig 3). More specifically, it involves linear regression of the sizes and their mutual correlation along with the corresponding *p*-values (showing if this correlation is random or not). The areas are represented as percentages of the informative area to the total image area (the same for all samples, i.e. 1200x1200 pixels). In the case of minced meat, the regression of the informative area sizes exhibits a line with 1.04 gradient and an offset of ~ -0.02 which indicates that we have a one to one relationship for the two methods. R-square, is just ~0.51, but it can be justified by the limited range of sizes (about 10% of the [0,1] range–from 0.62 to 0.72), preventing a good fit of a linear equation. Since the range of all samples are very close due to that their sizes are only affected by the fat they comprise, the fit of a line to data that forms an ellipsoid close to a disk shape can justify the relatively low R-square value. Another argument is that R-square shows the percentage of the data variance explained by the model. Having this in mind, we will try to explain its low value later via colocalization analysis. On the other hand, sizes’ correlation is high enough to further support the linear fit, exhibiting a correlation coefficient of >0.7 with *p*-value of ~5 10^−9^.

For the beef fillet, the slope of the fitted line is ~0.9 with an offset of 0.05 and an R-square value about 0.95. The correlation on the other hand is smaller than in the case of minced meat, ~0.55 with *p*-value ~ = 5 10^−5^. From Table 1 one can wonder on the low correlation coefficient *r* in contrast to high R-square value. This can be explained by examining the scatter of data points in Fig 3. As someone can notice there are several data points exhibiting a rather anti-correlation, i.e. small (large) sizes detected by VM and for the same image, large (small) area sizes in the case of AS. Those points are the most distant from the regression line and are the ones responsible for the low correlation coefficient value. On the other hand, R-square is not affected since those points are lying at both sides of the regression line and thus the goodness of fit is high. Finally, the regression of the pork fillet samples exhibits a slope of ~1 (again one to one relationship) with an offset of ~0.009 and R-square about 0.95. Additionally, we also have in this case a strong correlation (~0.75 and *p*-value ~ 2 10^−9^).

**Table 1:** Regression Analysis. α, parameter α of the linear regression model (*f(x) = ax + β*); β parameter β of the linear regression model; R-square, goodness of fit; RMSE, root mean square error; r, correlation coefficient; *p*-value, the *p*-value of the hypothesis that correlation between values are random.

	Α	Β	R-square	RMSE	r	*p*-value
**Minced Meat**	1.04	-0.02	0.51	0.04	0.72	5 10^−9^
**Beef Fillet**	0.90	0.05	0.95	0.02	0.54	4.8 10^−5^
**Pork Fillet**	1.00	0.01	0.97	0.01	0.75	2.1 10^−9^
**Table Olives**	1.06	-0.01	0.97	0.002	0.99	1.4 10^−15^
**Vanilla Crèmes**	0.59	0.11	0.22	0.005	0.24	0.31

In the case of vanilla crème samples we can say that the regression of size of the informative areas performed very poor since we do not have the one to one relationship (0.59 slope with R-square ~0.22) nor a high degree of correlation (correlation coefficient ~0.24 not statistical significant, *p*-value ~.31). As we will see later at the colocalization analysis, this is not due to the fact that the detected areas are not more or less the same but due to the regression method itself since the range of areas is very limited (the difference in sizes is about 0.03% normalized to image’s size and the size of all the samples are the same due to the production procedure). So, the data forms a small cloud around one value (~0.65%) with small variance where a line is very limited to be fitted (please refer to Fig 3(E)).

Finally, for the table olive samples, the linear regression exhibits a clear one to one relationship, i.e. the slope of the fitted line is 1.06 with R-square value of 0.97. Additionally, the correlation results in a coefficient about 0.99 supported with a *p*-value of 1.4 10^−15^. It is evident at this point, that the analysis of the informative area sizes, shows that the AS method is efficient and robust when compared to a laborious and human dependent analysis of VM.

**Fig 4:**
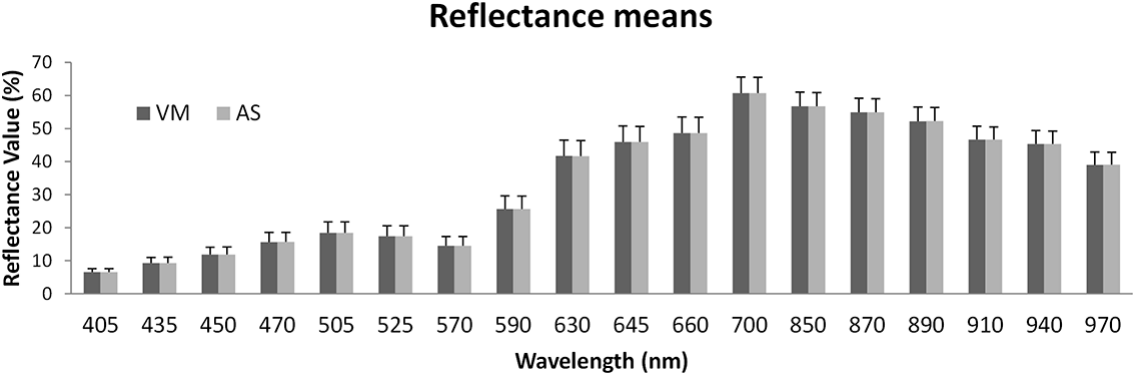
Analysis of mean reflectance values of the detected informative area at each wavelength and their corresponding standard deviations for minced meat dataset. It is obvious that all values are almost identical, i.e. we get the same information by either method, AS or VM.

In order to further strengthen the outcomes of the previous analysis and also explore additional aspects that have practical applications for research scientists, we also conducted an analysis of the mean reflectance values of the informative areas and their corresponding standard deviations (please refer to Fig 4). In order to assess this information we present in Fig 4 the bar plot for the minced meat dataset (S3 File holds the bar plots for beef and pork fillet datasets along with the table showing the numeric results for comparison), where the bars show the mean reflectance values and the error bars the corresponding standard deviations. It is more than clear that the outcomes are almost identical. Performing a correlation analysis on those data (refer to the tables in S3 File) it is evident that the values measured are almost identical since all correlation coefficients verge to 1 and *p*-values to 0 (10^−19^ the highest value). Taking this into consideration along with the previous analysis, we can argue that the two methods perform equally. In addition, the argument that the informative area extraction has been performed manually by an expert user with VideoMeterLab software, we can state that the developed, fully automated workflow enables high throughput analysis of multispectral food images efficiently.

**Fig 5:**
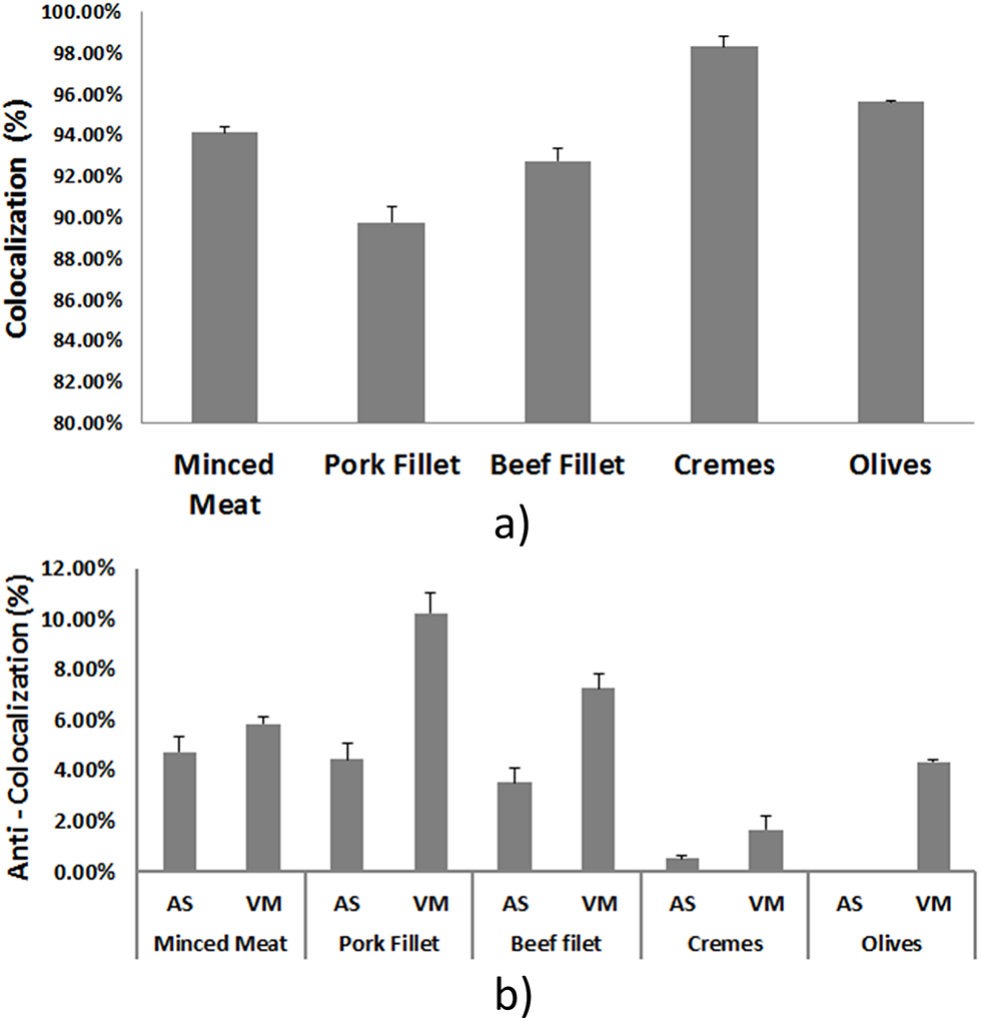
Colocalization Analysis. (a) mean colocalized areas for the three datasets on the basis of VM’s detected informative area; error bars represent the standard error of the colocalization, (b) “Anti-colocalization” percentages means and standard error (anti-colocalization stands for areas that have not been detected by the other method).

To further investigate the results and search for any inconsistencies between the detected informative areas, we performed colocalization analysis. Colocalization is a common and popular methodology in biosciences for measuring the degree of overlapping fluorescence of fluorescent proteins. In general, this analysis concerns not only the binary question: is the fluorescent dyes colocalized or not; but also the intensity of fluorescence. In our case, the intensities have no useful information since they do not provide insight for the dyes. So, the analysis is based only on the binary question of whether or not, AS informative areas overlap with the ones detected by VM, and at what percentage. Fig 5(A) presents the mean percentage of colocalization for each dataset along with the respective Standard Error (SE). Additionally, from Fig 5(A), it should be noticed that we have a high degree of colocalization, >90% with small SE; pork fillet dataset found to have the lowest colocalization, but this should not be considered as a method’s inefficiency, as it was shown from the previous two analyzes’ results. Further, it should be noted, that the percentages are computed using VM results as the basis (“ground truth”, i.e. we compare the AS to the VM areas). Next, Fig 5(B) presents the percentages (again under the assumption of VM results as the “ground” truth) of the areas that were detected only by the AS method and not by the VM method and vice versa (i.e. the percentages of mutually exclusive areas compared to VM). As expected, the results reflect the ones shown in Fig 5(A). An interesting observation, most obvious in the “worst” case (pork fillet), but consistent across all five datasets, is that the VM contributes more than the AS "exclusive" areas to the relatively low colocalization percentage (>90%). Table 2 holds the numeric values of Fig 5, provided as reference. At this point we must elaborate on the regression results for the vanilla crème samples. As we presented earlier the linear regression was not able to show a one to one relationship in this case. On the other hand the colocalization analysis shows that for these samples we get the highest degree of colocalization (>98%). These outcomes, coupled with the rational of a small variance round a fixed number (true sample size ~0.65%), justify our assumption that the linear regression fails to result in a good fit.

**Table 2:** Colocalization analysis. mean colocalization; Standard Error (SE);

	Minced Meat		Pork Fillet		Beef filet		Crèmes		Table olives	
Mean	94.14%		89.77%		92.75%		98.32%		95.64%	
SE	0.27%		0.78%		0.58%		0.53%		0.05%	
	AS	VM	AS	VM	AS	VM	AS	VM	AS	VM
Mean	4.74%	5.86%	4.45%	10.23%	3.55%	7.25%	0.53%	1.68%	0.00%	4.36%
SE	0.61%	0.27%	0.63%	0.78%	0.55%	0.58%	0.12%	0.53%	0.00%	0.05%

The aforementioned colocalization analysis results need further investigation in order to infer them. Due to lack of space, the additional figures are supplied in the form of S4 File, but the findings will be discussed here. After careful visual investigation we came to the conclusion that VM excludes erroneously several informative areas mainly at the contour of the sample. This holds for all the images processed with VM, being justifiable by two reasons: a) the over-specification of the segmentation model built by the user’s manual annotation (informative areas, surrounding, fat/connective tissues) and b) the user’s subjectivity at the labeling process. Just because the developed methodology does not lean on any of those limitations, the informative area extraction can be safely considered more robust and efficient. Furthermore, via this analysis we identified a parameter explaining partially the observed non perfect colocalization. This also reflects to the non-perfect one to one relationship between the sizes of the informative areas extracted by VM and AS (as discussed earlier). In order to fully explain the imperfect colocalization and linear regression results, we also need to look at the AS only extracted areas (please refer to S4 File). Here, we can see that AS tends to overestimate the areas near the fat and connective tissues (for meat samples). This cannot be safely categorized either as error or not, because the user relies on specific spectral bands to observe and label the fat/connective tissue areas. The issue here is that different spectral bands show different evidence on whether a specific region is fat or not. So, what can be done at this point, in order to characterize this inconsistency, is that AS method process every image the same way, i.e. is biased to overestimate fat regions while VM is subject to the user’s intuition (overestimate on some, underestimate on other). Thus, AS is a reproducible method while VM not, simply because it requires human intervention. In the case of vanilla crème and table olive samples, the same conclusions hold. In both cases VM tends to overestimate the informative areas at their contours, while also detects several small scattered areas outside the informative areas.

The evaluation of the presented segmentation method, proves that it is a robust, efficient and reliable alternative to the semi-manual method of VideoMeterLab, in terms of informative area detection. Additionally, it is more time efficient. More specifically, VM requires (i) a series of images under different conditions (in order to capture sample variations) and (ii) a large amount of effort and trials for area annotation. Additionally, the user should look through the whole dataset and define the most “representative” image that includes all the different area states of the sample, label a large amount of area on the sample (meat, surrounding and fat/connective tissue), and then process the images using the model that has been built. It should be noted that in the case of VM, the processing time including user’s effort, for the processing of the 50 samples of each dataset took ca. 5 minutes for every image, while for the developed methodology, took about 5 minutes for the whole dataset. Finally, taking under consideration the subjectivity of the user, the informative areas result for VM, is not reproducible. In contrast, the proposed AS approach, requires no user intervention, is data driven and performs equally if not modestly better (concerning fat/connective tissue detection and exclusion) in all cases. The AS method is developed in MatLab environment in the form of a user friendly GUI which runs under Windows 7 x64 OS. The developed GUI can easily be compiled to run on other operating systems.

## Conclusions

An automated, high throughput multispectral image processing methodology for food data sample analysis, focused mainly to meat and meat products has been presented. It has been shown that the proposed method can be applied to other types of food, like olives and crèmes with the same observations hold. In fact this process “frees” the results from user’s bias and errors, while it has been proven to be robust considering the variation of the food sample inserted in the used datasets.

This analysis can be considered as the first but a crucial step towards automated, non-invasive analysis and time critical monitoring of food quality. Such a process could have been proven to have great impact in food industries as it enables the sampling of 100% of the products with very low cost and time efficiency, something that cannot be done with the current methodologies related to evaluation of food quality.

Future work includes a larger scale validation of the methodology on more food samples and further optimization of the processing time. The development of smart “diagnostic” methods that fuse machine learning and statistical schemes (of high-speed data processing and reduction) with tracking data to identify robust multiple fingerprints quality indices, is within the activities of our group and currently is in progress.

## Supporting Information

S1 File Figures of wavelength selection. This supplementary information file illustrates the EBCM based selection of wavelengths for the image segmentation process.
(PDF)

S2 File Pseudo RGB image acquired by VideoMeterLab. Example of several sample images.
(PDF)

S3 File Analysis of mean reflectance values for beef and pork fillet datasets. Supplement to Fig 4.
(PDF)

S4 File Colocalization Analysis. This file contains in more detail the colocalization analysis applied.
(PDF)

S5 File Data for regression, correlation and statistical analysis. (ZIP)
