# Supplementary material for: High Throughput Multispectral Image Processing with Applications in Food Science
Source: PLoS One. 2015 Oct 14;10(10):e0140122. doi: 10.1371/journal.pone.0140122 (PMC4605757; doi:10.1371/journal.pone.0140122)

Pseudo RGB image of the Raw Image

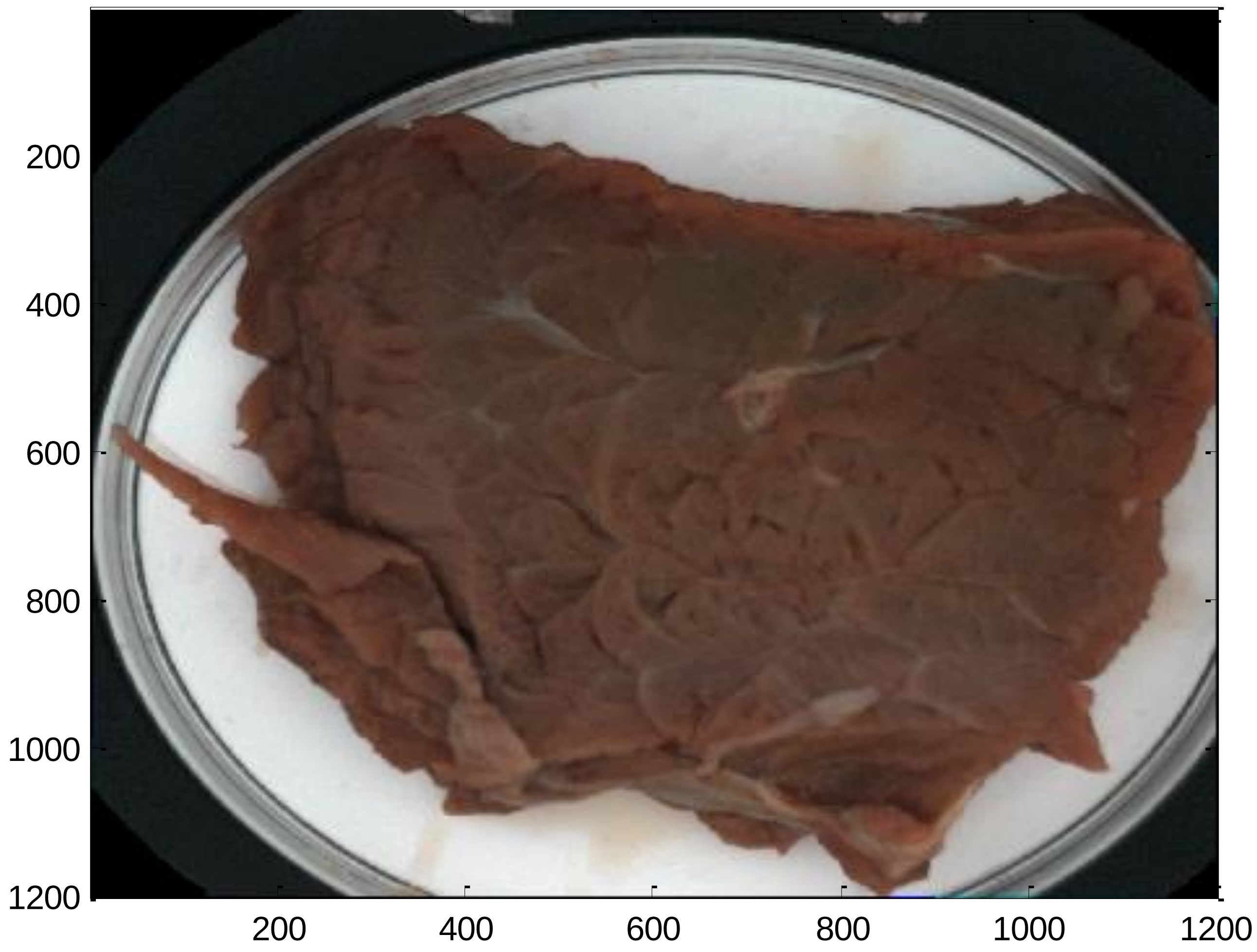

Combination of images at wavelengths 405nm and 590nm. This combination exhibited the lowest EBCM value and distinguish better the surrounding from the meat sample and petri dish

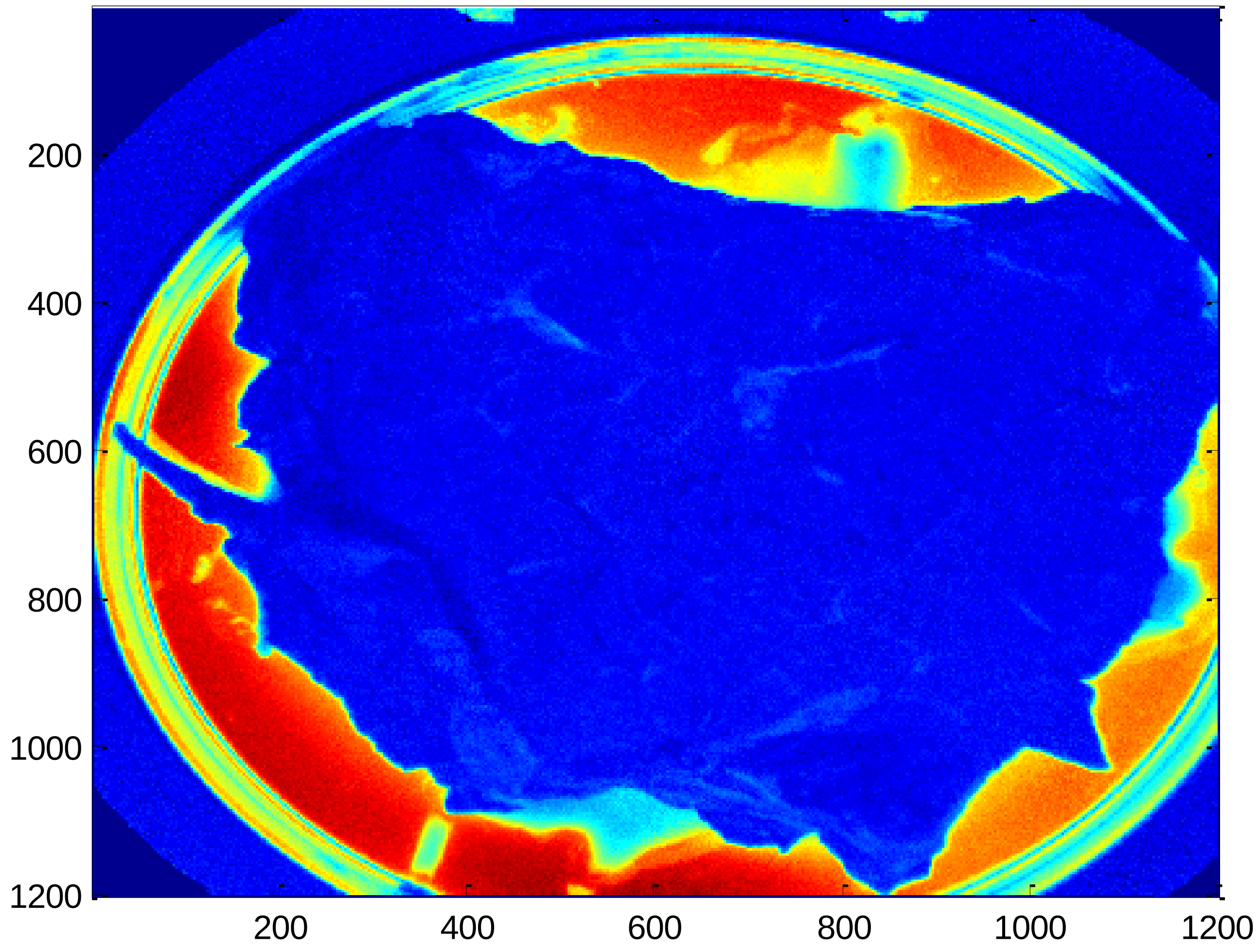

Combination of images at wavelengths 405nm and 435nm. This combination exhibited the lowest EBCM value and distinguish better the meat areas from fat and connective tissue.

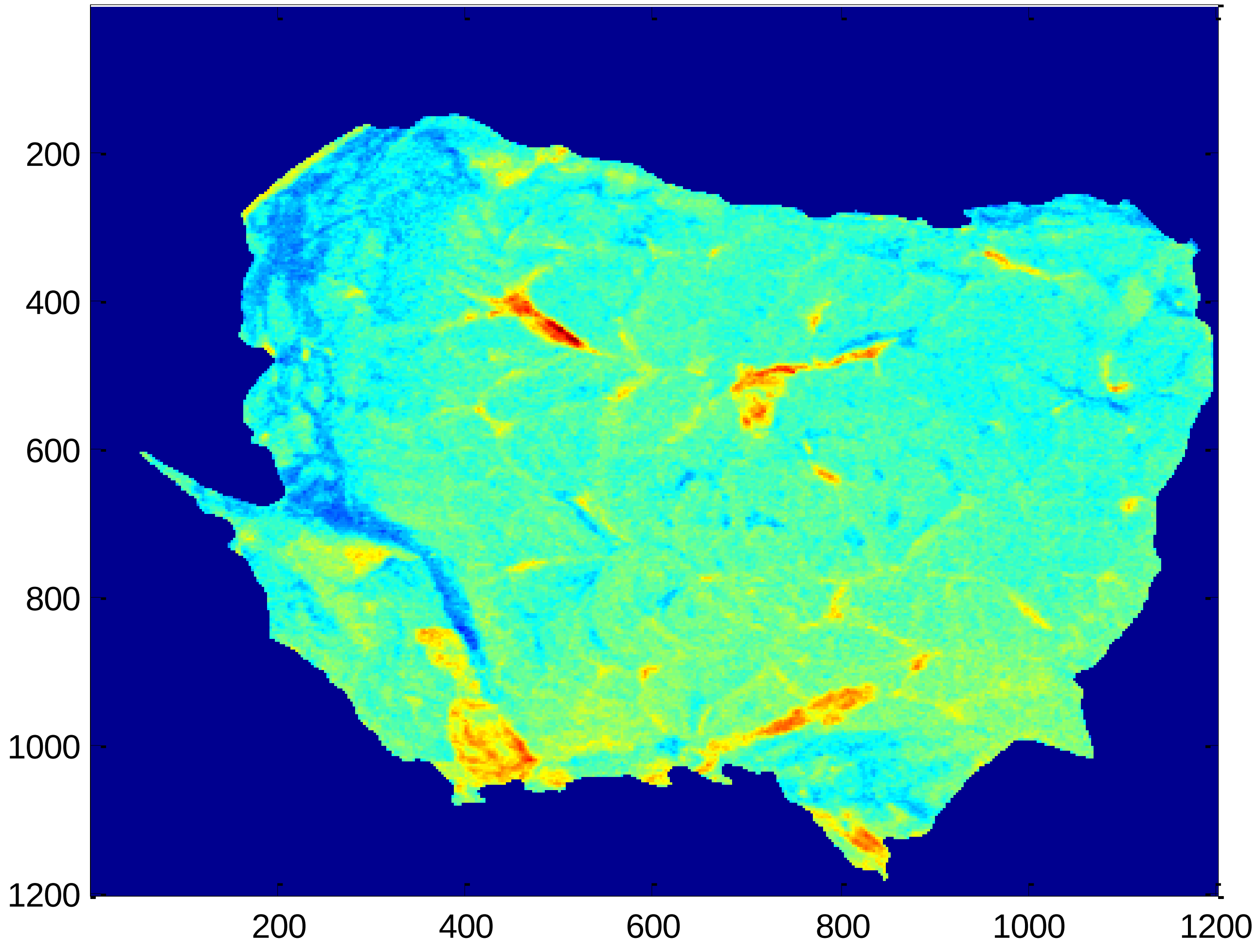

Final Segmented image, only meat areas!

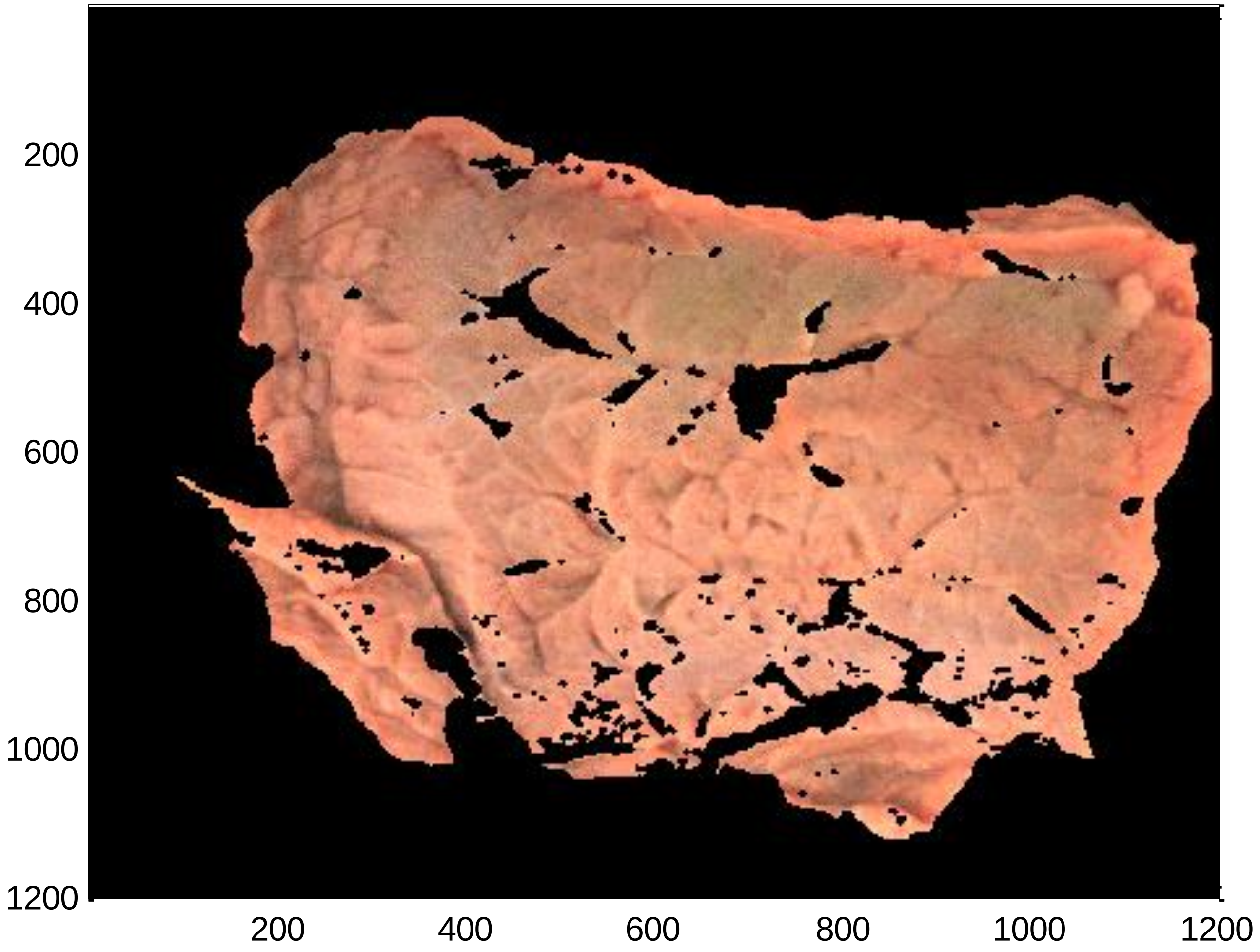

Supplement: S1 File — This supplementary information file illustrates the EBCM based selection of wavelengths for the image segmentation process. (PDF) [file pone.0140122.s001.pdf]
