# Supplementary material for: High Throughput Multispectral Image Processing with Applications in Food Science
Source: PLoS One. 2015 Oct 14;10(10):e0140122. doi: 10.1371/journal.pone.0140122 (PMC4605757; doi:10.1371/journal.pone.0140122)
Supplement: S2 File — Example of several sample images. (PDF) [file pone.0140122.s002.pdf]

Supplementary Information 2 (SI2)

Images Acquired by VideometerLab

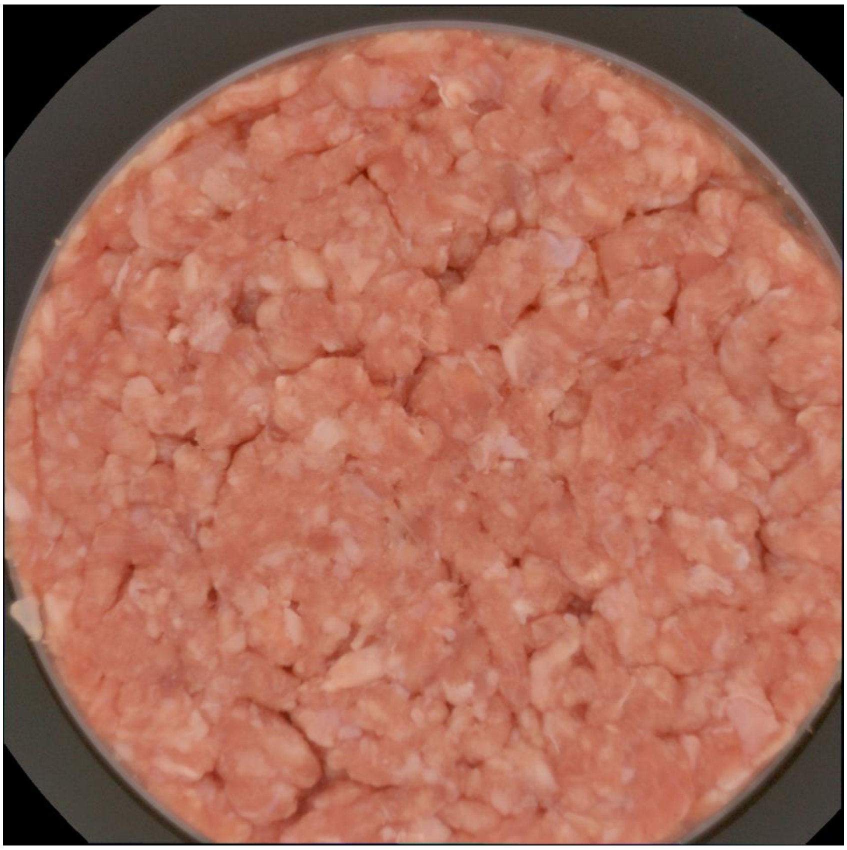

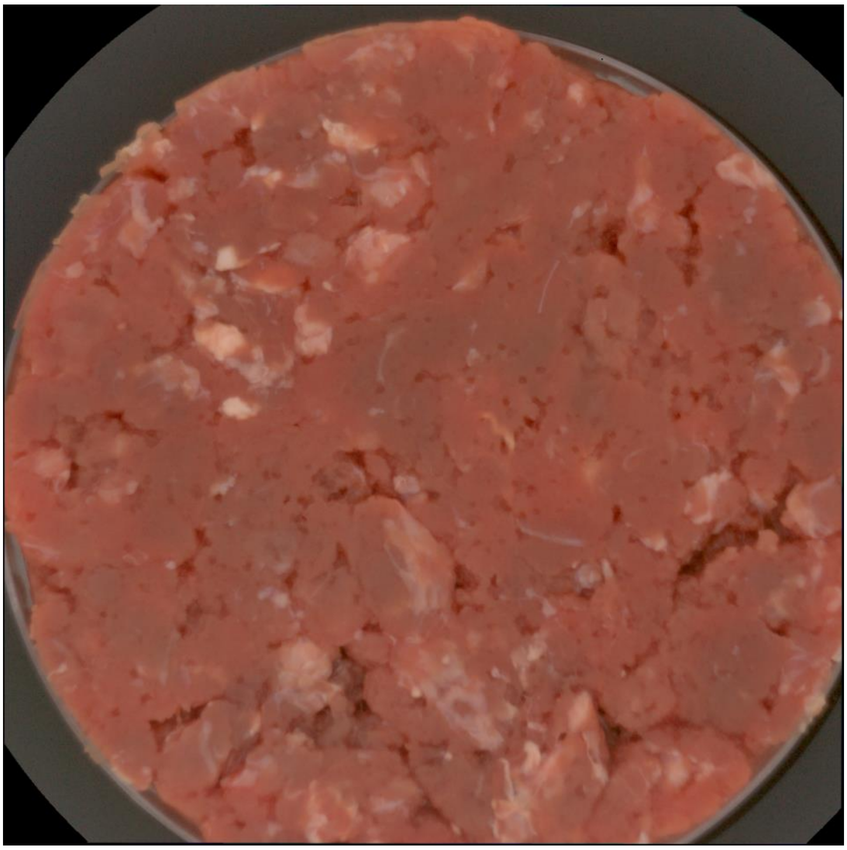

Figure SI2.1. Minced Meat Images.

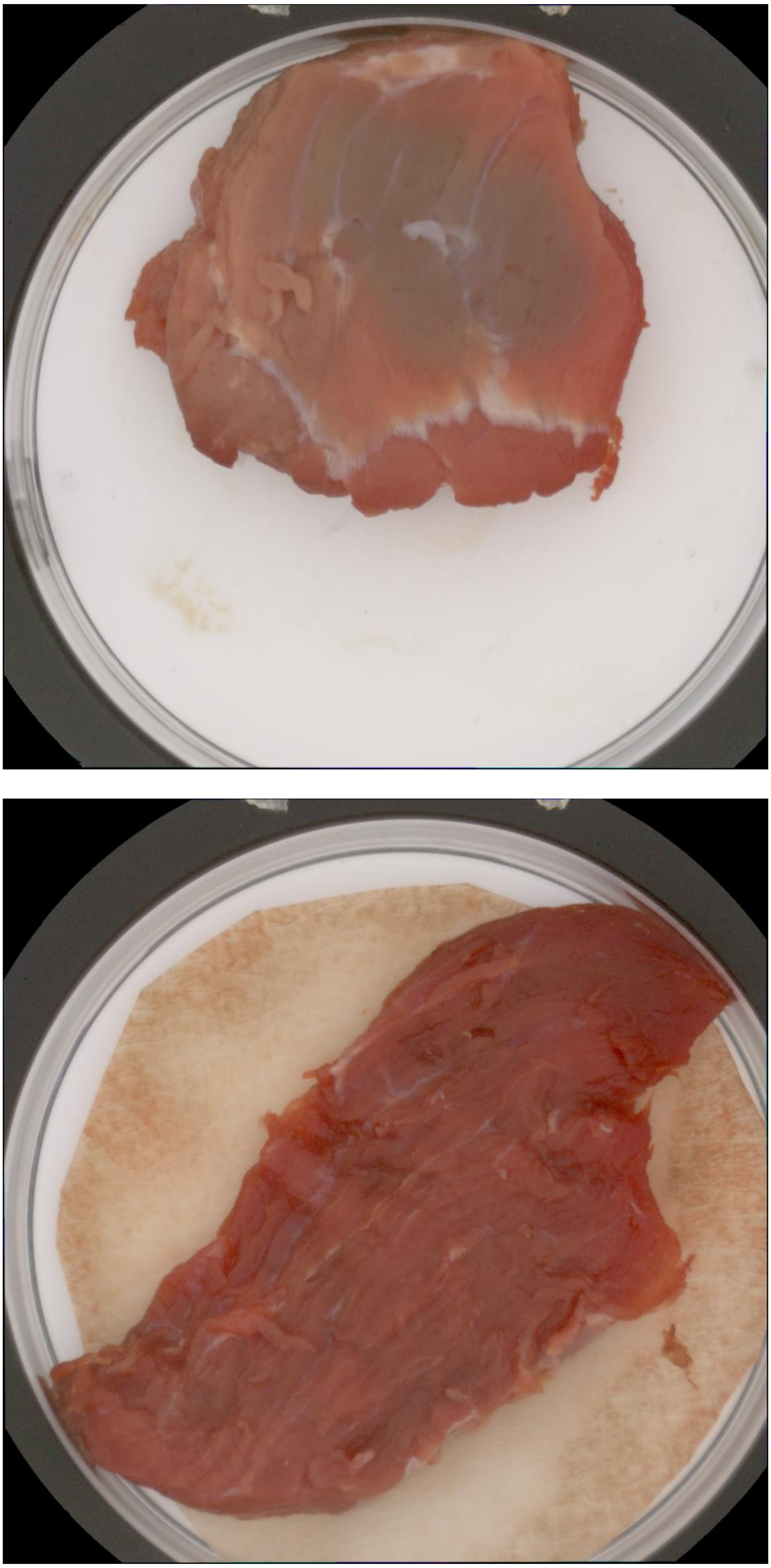

Figure SI2.2. Beef Fillet Images.

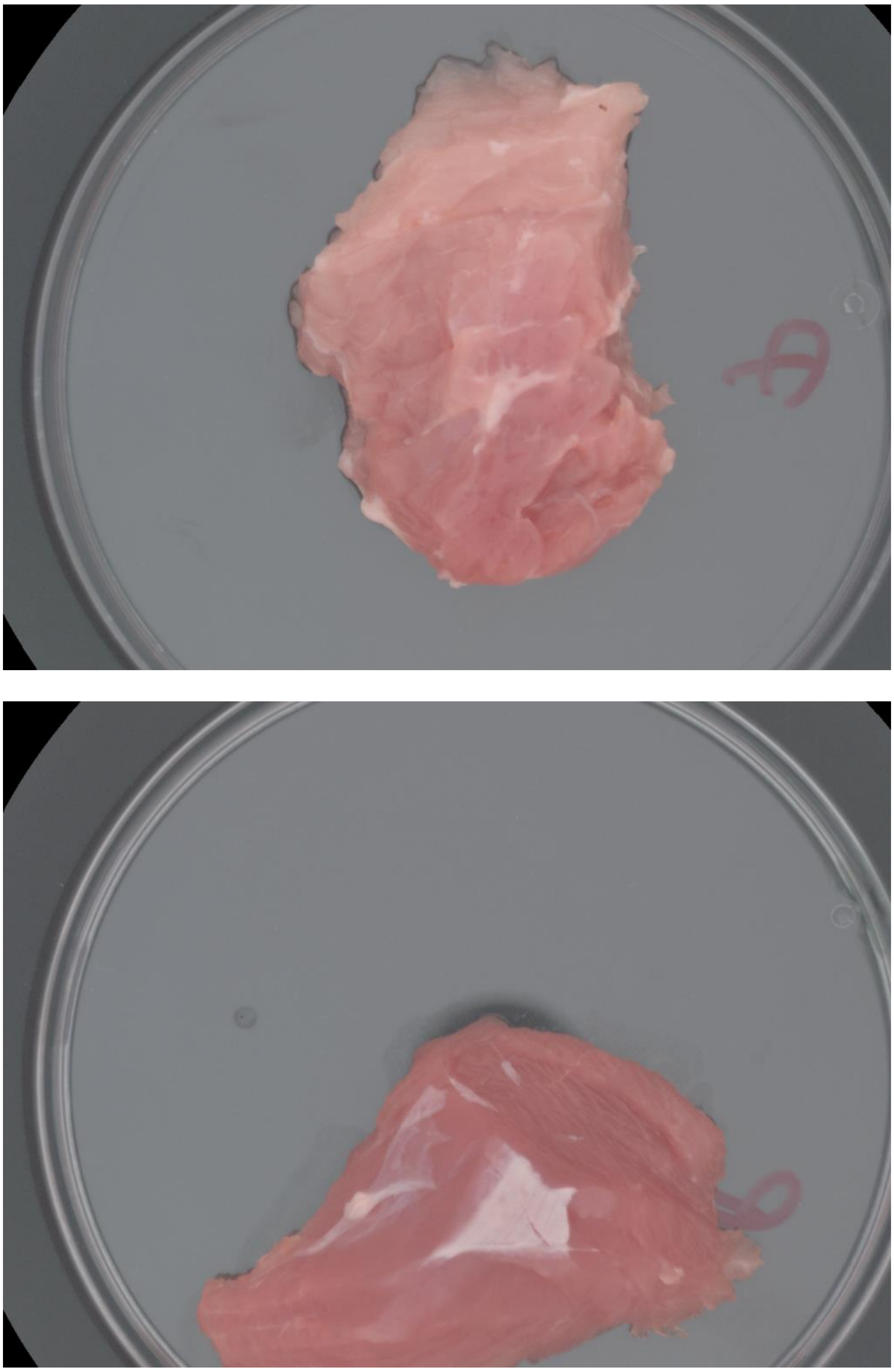

Figure SI2.3. Pork Fillet Images.

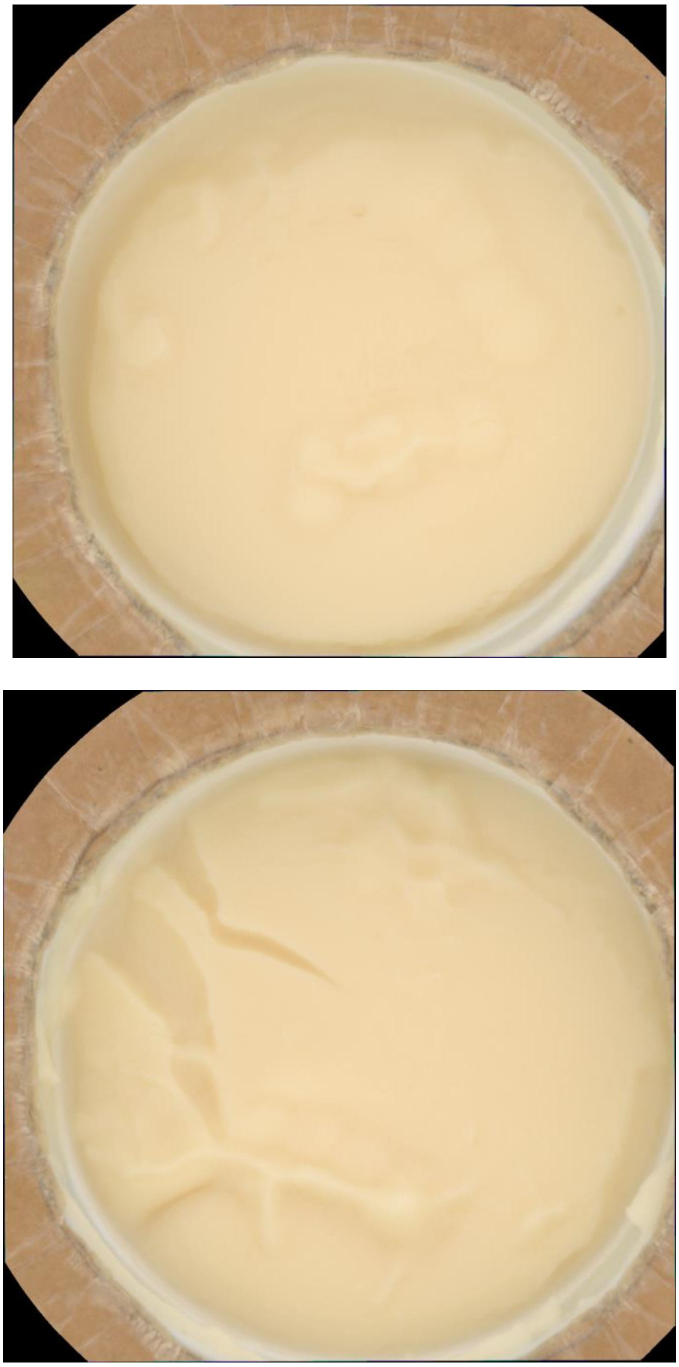

Figure SI2.4. Crèmes Images.

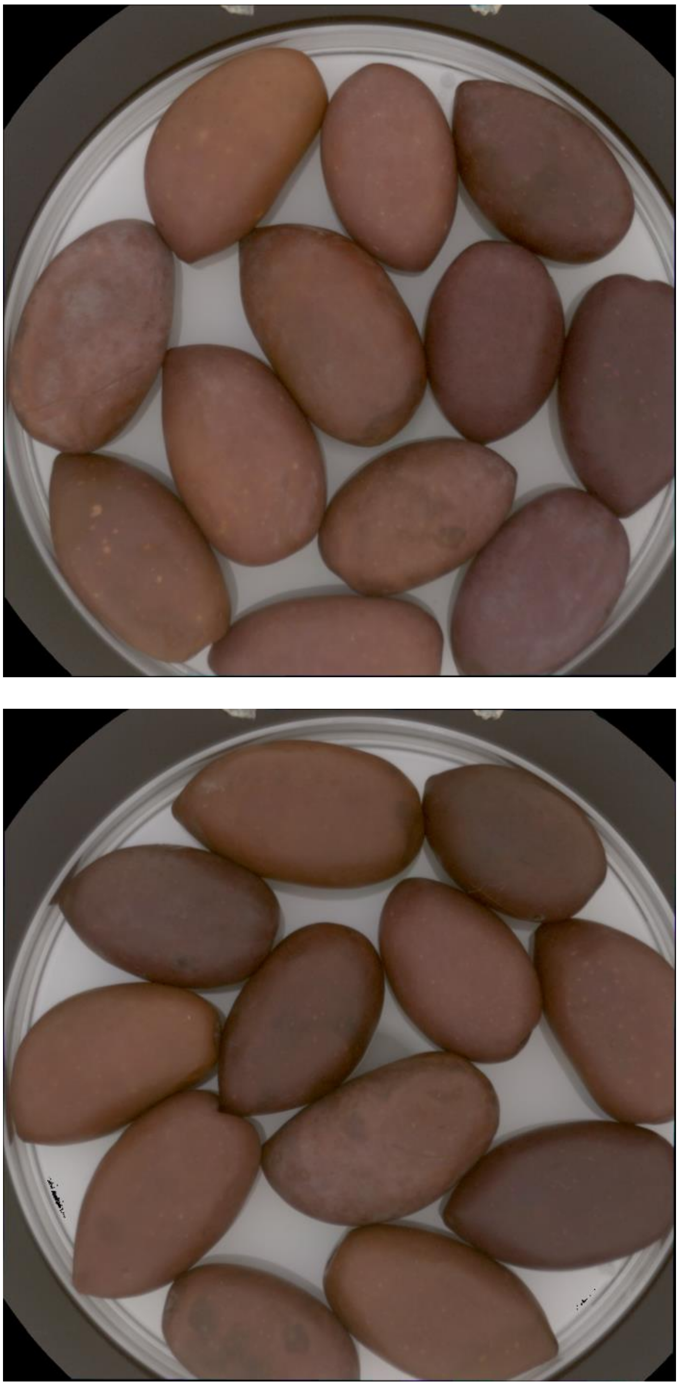

Figure SI2.5. Table olives Images.
