# Supplementary material for: High Throughput Multispectral Image Processing with Applications in Food Science
Source: PLoS One. 2015 Oct 14;10(10):e0140122. doi: 10.1371/journal.pone.0140122 (PMC4605757; doi:10.1371/journal.pone.0140122)
Supplement: S3 File — Supplement to Fig 4. (PDF) [file pone.0140122.s003.pdf]

Supplementary Information 3 (SI3)

Mean Reflectance Values Analysis

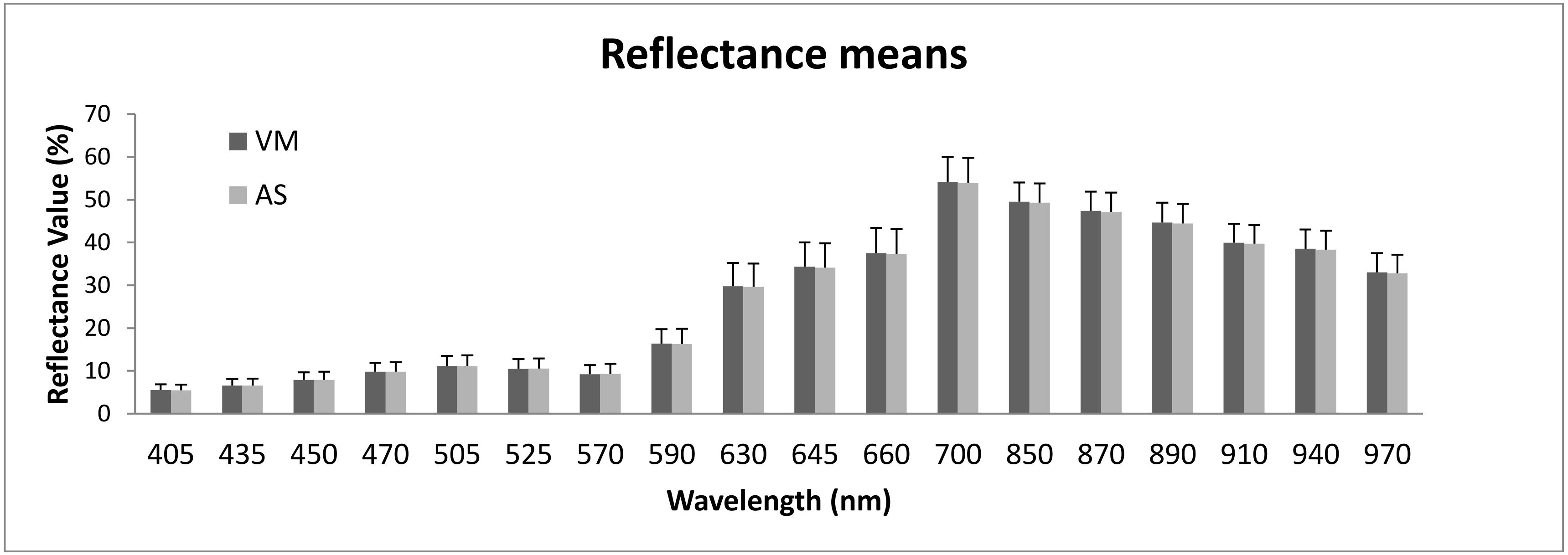

Figure SI3.1. Beef Fillet. Analysis of mean reflectance values of the detected informative area at each wavelength and their corresponding standard deviations for minced meat dataset. It is obvious that all values are almost identical, i.e. we get the same information by either method, AS or VM.

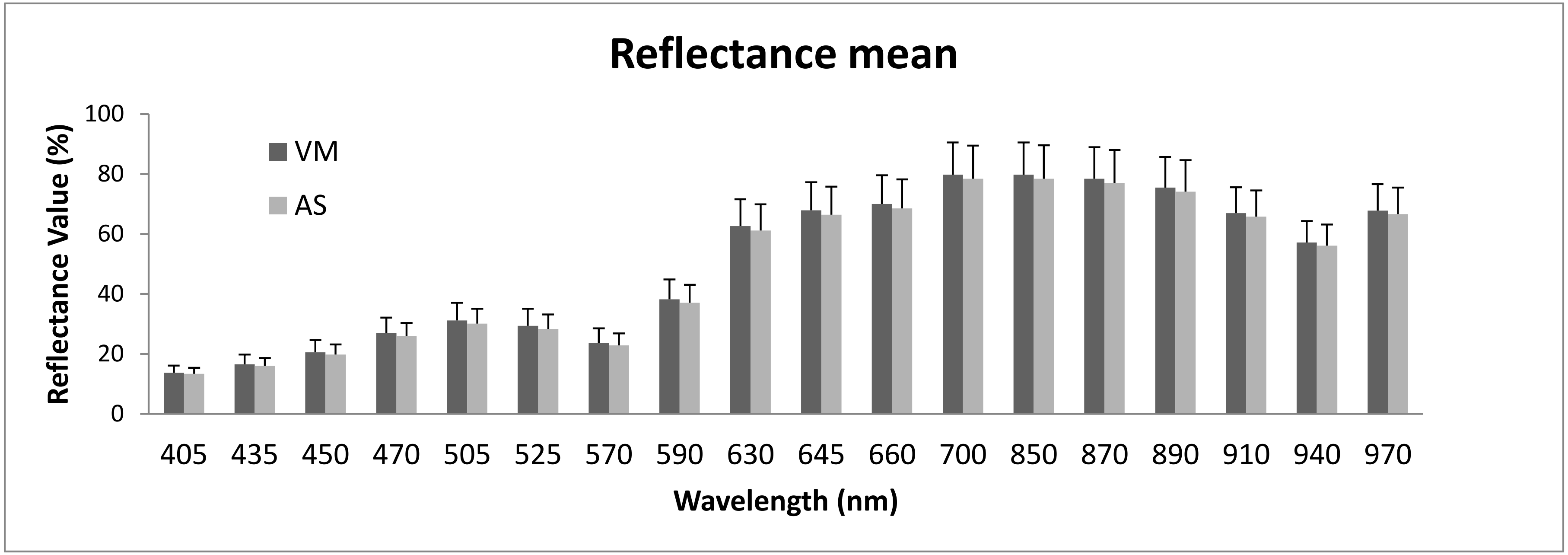

Figure SI3.2. Pork Fillet. Analysis of mean reflectance values of the detected informative area at each wavelength and their corresponding standard deviations for minced meat dataset. It is obvious that all values are almost identical, i.e. we get the same information by either method, AS or VM.

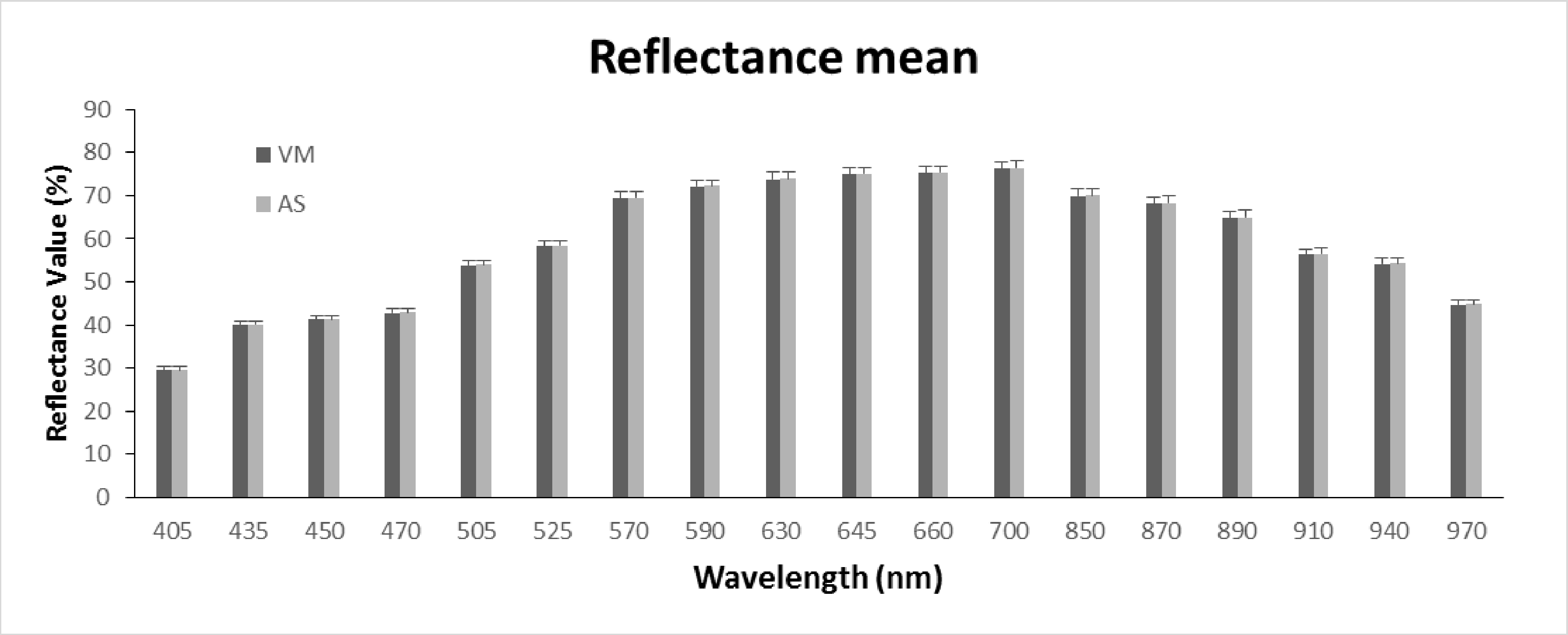

Figure SI3.3. Crèmes. Analysis of mean reflectance values of the detected informative area at each wavelength and their corresponding standard deviations for crèmes dataset. It is obvious that all values are almost identical, i.e. we get the same information by either method, AS or VM.

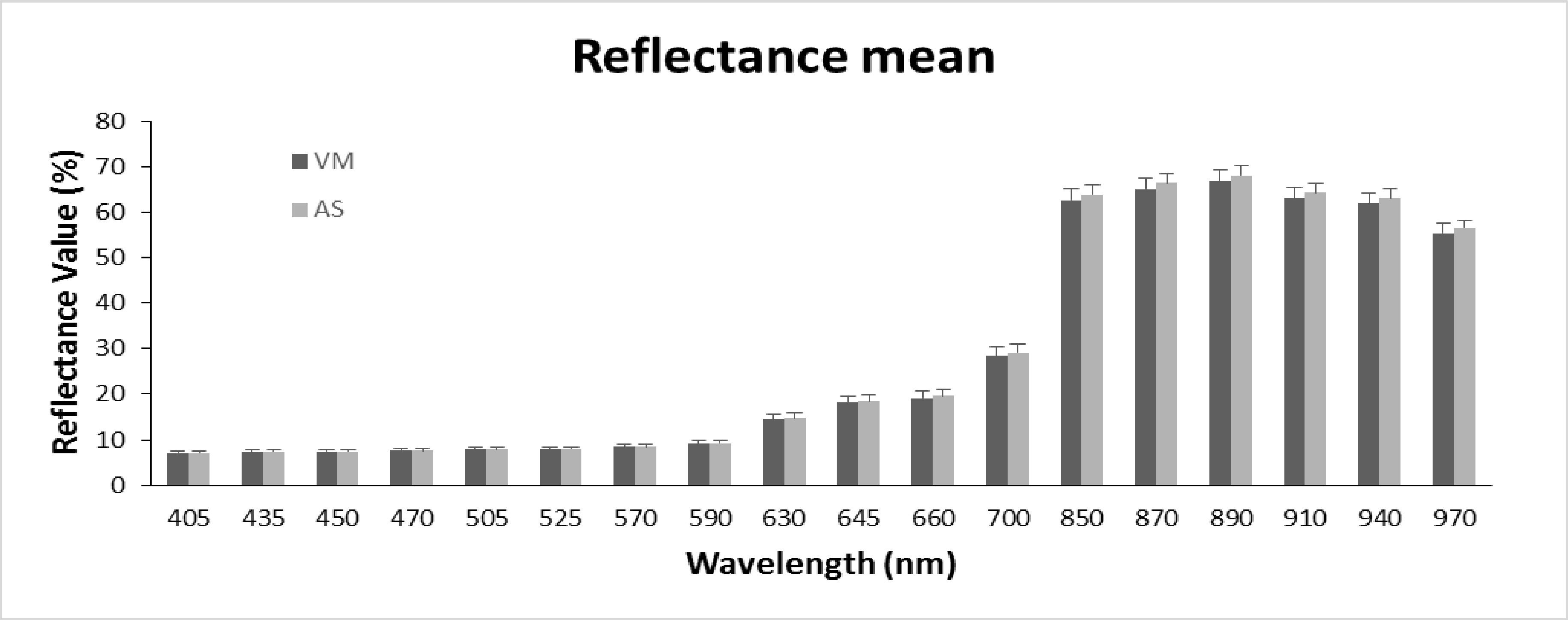

Figure SI3.4. Table Olives. Analysis of mean reflectance values of the detected informative area at each wavelength and their corresponding standard deviations for table olives dataset. It is obvious that all values are almost identical, i.e. we get the same information by either method, AS or VM.

	Minced Meat		Beef Filet		Pork Filet	
WaveLength (nm)	Correlation Coefficients	p - Values	Correlation Coefficients	p - Values	Correlation Coefficients	p - Values
405	0.997294832	4.40007E-56	0.977775763	3.16602E-34	0.988270765	9.86499E-37
435	0.99672451	4.31212E-54	0.962258113	8.81024E-29	0.991780223	4.88975E-40
450	0.99710043	2.32205E-55	0.96084839	2.09089E-28	0.992023822	2.56699E-40
470	0.997661578	1.33879E-57	0.965822748	8.47799E-30	0.992119833	1.98042E-40
505	0.998531032	1.92765E-62	0.964350989	2.29387E-29	0.992427652	8.43367E-41
525	0.998448978	7.09853E-62	0.961460412	1.44244E-28	0.992492495	7.01443E-41
570	0.997840073	1.99505E-58	0.942663768	1.61236E-24	0.99264911	4.46491E-41
590	0.999309802	2.60516E-70	0.961108755	1.78672E-28	0.993159812	9.54218E-42
630	0.999783057	2.26476E-82	0.966519909	5.21046E-30	0.990278624	1.77635E-38
645	0.999807219	1.33163E-83	0.96828932	1.44392E-30	0.988404984	7.7127E-37
660	0.999806262	1.49975E-83	0.969839954	4.41084E-31	0.987250658	5.86708E-36
700	0.999714534	1.64328E-79	0.984925445	3.08401E-38	0.983049925	2.56823E-33
850	0.999658211	1.23687E-77	0.957036078	1.86295E-27	0.968597584	1.27346E-27
870	0.99967555	3.54622E-78	0.953923044	9.64004E-27	0.968242785	1.61569E-27
890	0.999687613	1.42863E-78	0.950514013	5.14431E-26	0.967429029	2.76117E-27
910	0.999678825	2.77998E-78	0.940357296	4.04736E-24	0.974271484	1.85631E-29
940	0.999672626	4.39826E-78	0.934881639	3.13073E-23	0.989183947	1.74254E-37
970	0.999654322	1.62274E-77	0.903169731	2.97218E-19	0.972340848	8.62889E-29

Table SI3.1. Correlation analysis results. In any situation correlation coefficient tends to 1 with a *p*-value that is is not ramdom much less than 10^{-19}.

	Cremes		Table Olives	
WaveLength (nm)	Correlation Coefficients	p - Values	Correlation Coefficients	p - Values
405	0.994350739	5.45289E-19	0.999216084	1.05863E-26
435	0.997161606	1.12398E-21	0.999608287	2.05897E-29
450	0.997186399	1.03874E-21	0.999654941	6.57719E-30
470	0.996929012	2.28162E-21	0.999702043	1.75576E-30
505	0.994512008	4.20413E-19	0.999772324	1.55968E-31
525	0.993278533	2.59536E-18	0.999786726	8.66286E-32
570	0.98405207	5.98227E-15	0.99979839	5.22193E-32
590	0.976487987	1.91519E-13	0.999844308	5.10162E-33
630	0.963491792	9.58387E-12	0.99989663	1.2792E-34
645	0.98332608	8.90685E-15	0.999904408	6.32661E-35
660	0.954252044	7.05786E-11	0.999903427	6.93555E-35
700	0.935734726	1.40438E-09	0.999912012	3.00065E-35
850	0.928894826	3.40254E-09	0.999817058	2.17808E-32
870	0.987111017	8.8983E-16	0.99977133	1.62208E-31
890	0.935558117	1.43856E-09	0.999683028	3.06361E-30
910	0.946067244	3.01254E-10	0.999408103	8.44956E-28
940	0.950740863	1.35553E-10	0.999322396	2.85277E-27
970	0.969229992	2.10026E-12	0.998595954	2.00346E-24

Table SI3.2. Correlation analysis results. In any situation correlation coefficient tends to 1 with a *p*-value that is is not ramdom much less than 10^{-19}.
