# Supplementary material for: High Throughput Multispectral Image Processing with Applications in Food Science
Source: PLoS One. 2015 Oct 14;10(10):e0140122. doi: 10.1371/journal.pone.0140122 (PMC4605757; doi:10.1371/journal.pone.0140122)
Supplement: S4 File — This file contains in more detail the colocalization analysis applied. (PDF) [file pone.0140122.s004.pdf]

# Supplementary Information 4 (SI4)

# Colocalization Analysis

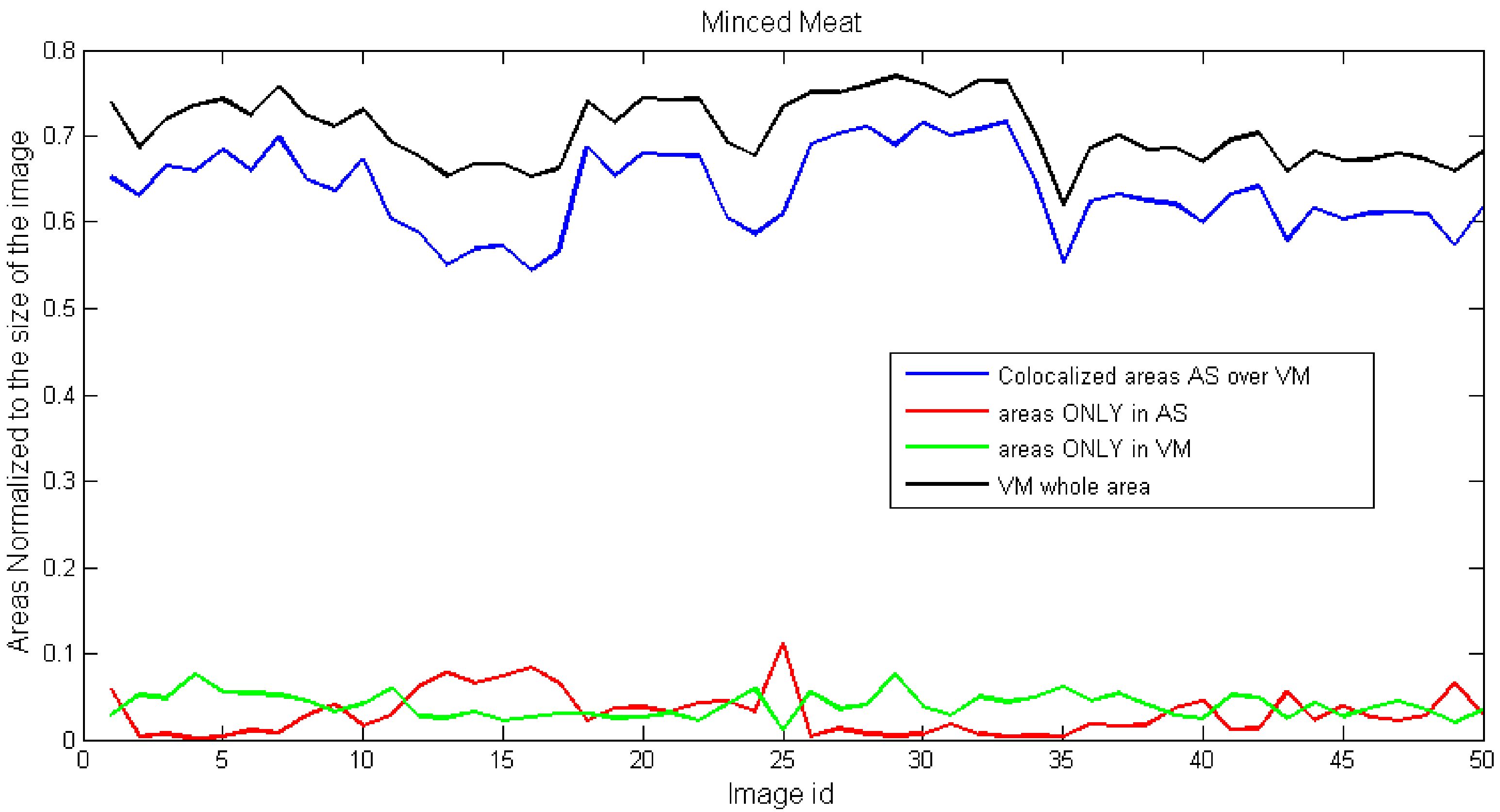


**Figure SI4.1. Minced Meat.** Areas Normalized to the size of VM area and shown as coverage percentage to the size of the whole image. Black line indicates the informative area detected by VM, blue line the colocalized areas of AS and VM. Green line the area of VM that do not colocalized to AS and red line the AS area not colocalized to VM.

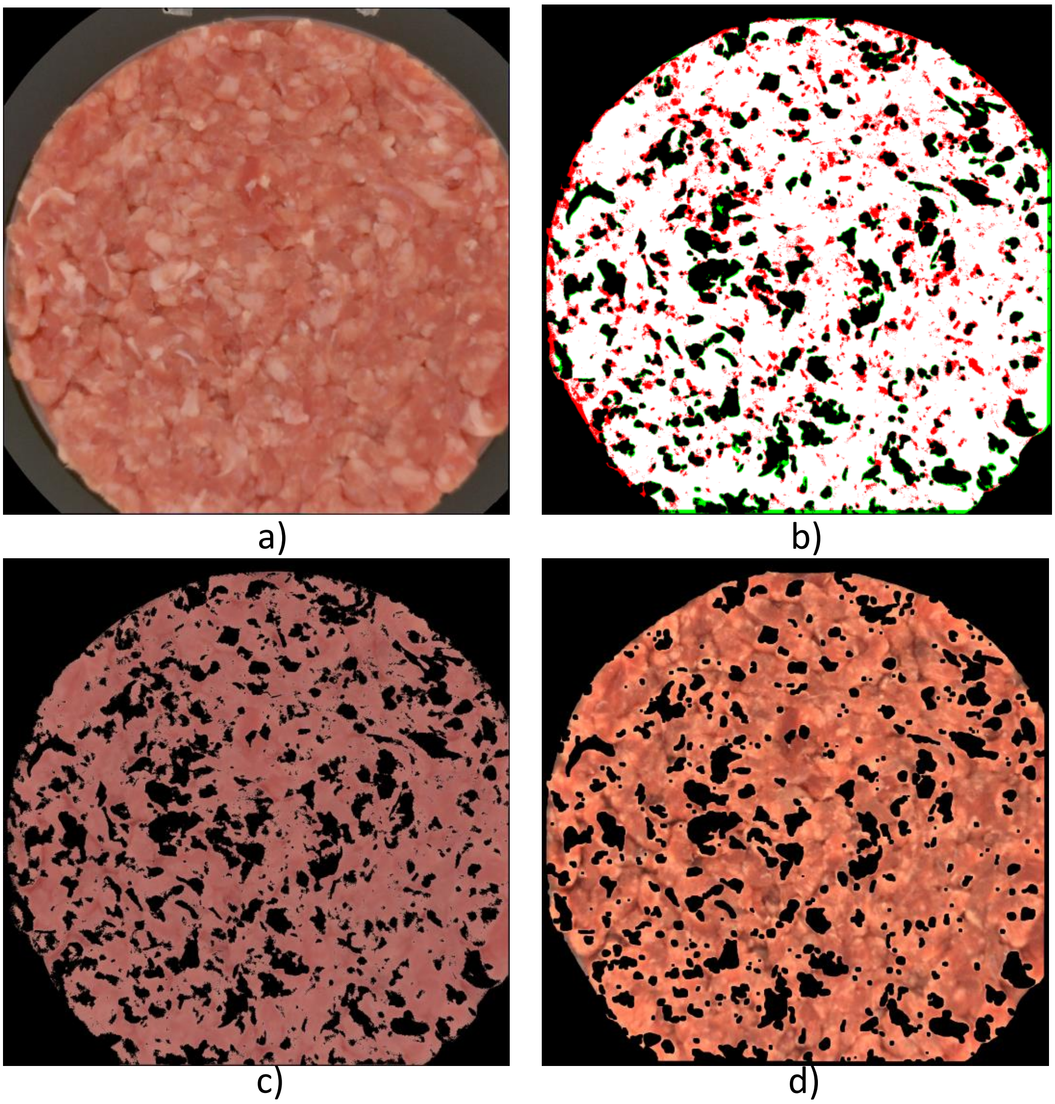


**Figure SI4.2. Minced Meat.** (a) original pseudo-RGB image, (b) colocalization of AS and VM areas shown in white, green regions indicate the areas detected only by VM, while red areas those that AS have detected, (c) VM informative area, (d) AS informative area.

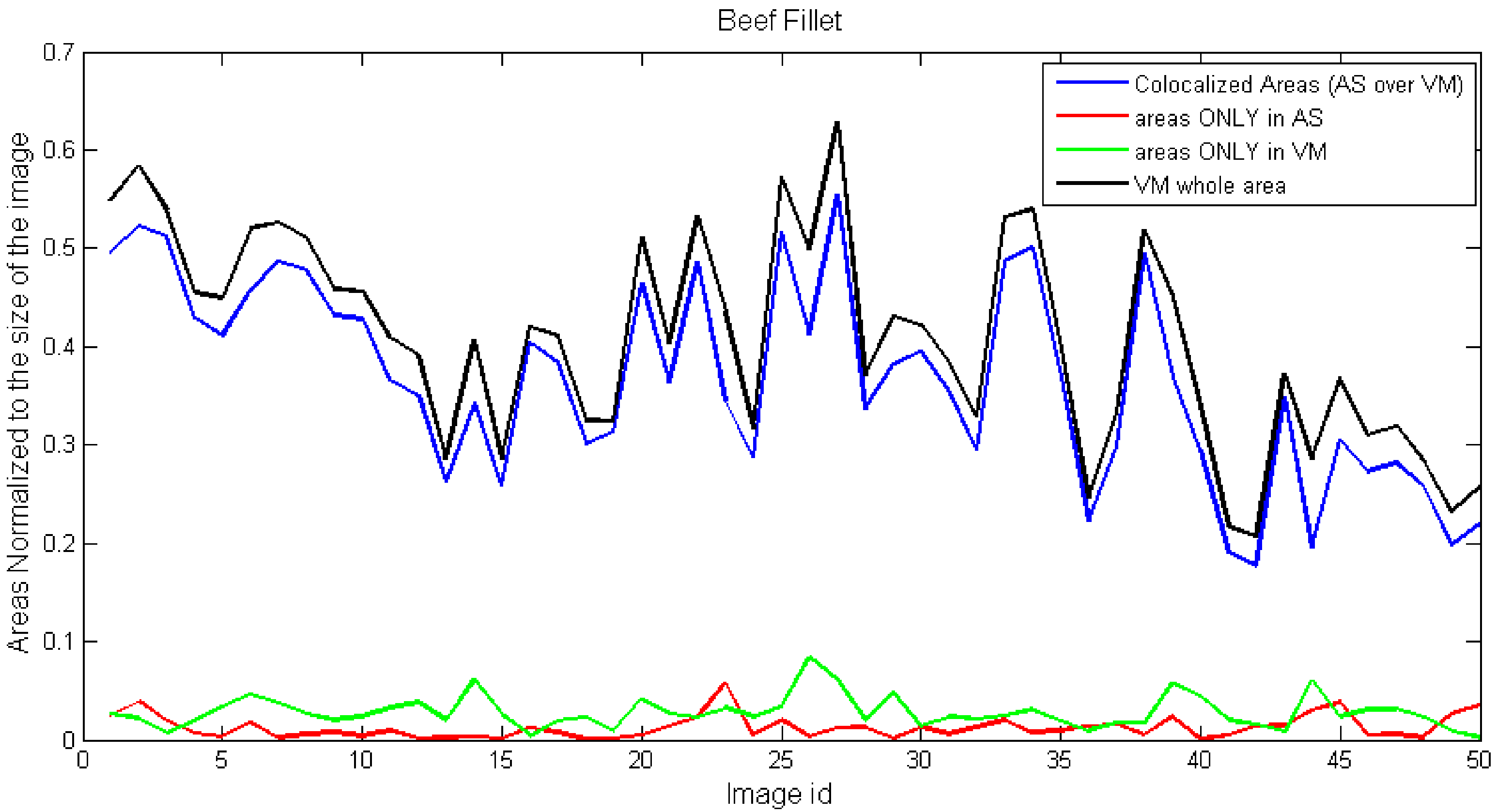


**Figure SI4.3. Beef Fillet.** Areas Normalized to the size of VM area and shown as coverage percentage to the size of the whole image. Black line indicates the informative area detected by VM, blue line the colocalized areas of AS and VM. Green line the area of VM that do not colocalized to AS and red line the AS area not colocalized to VM.

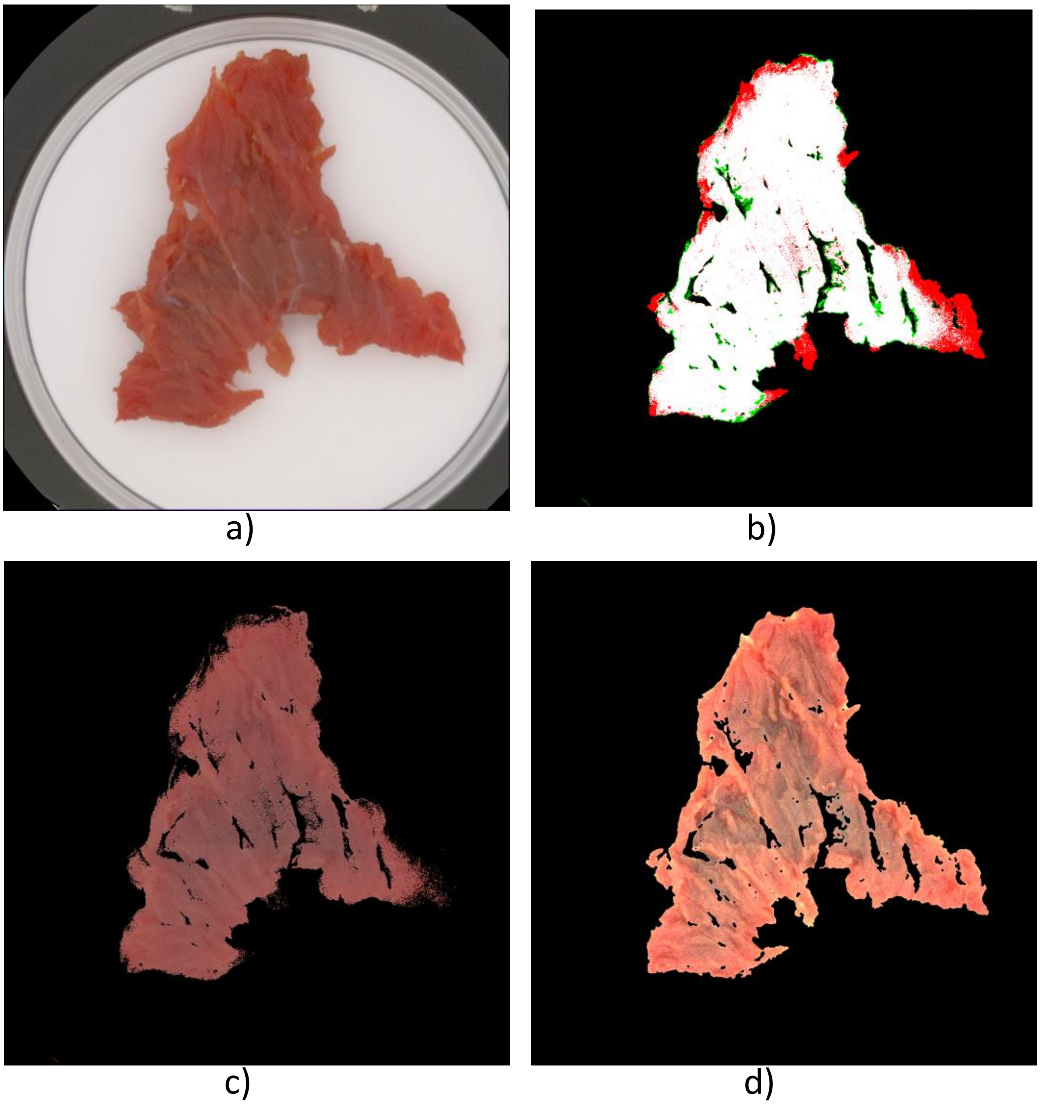

**Figure SI4.4. Beef Fillet.** (a) original pseudo-RGB image, (b) colocalization of AS and VM areas shown in white, green regions indicate the areas detected only by VM, while red areas those that AS have detected, (c) VM informative area, (d) AS informative area.

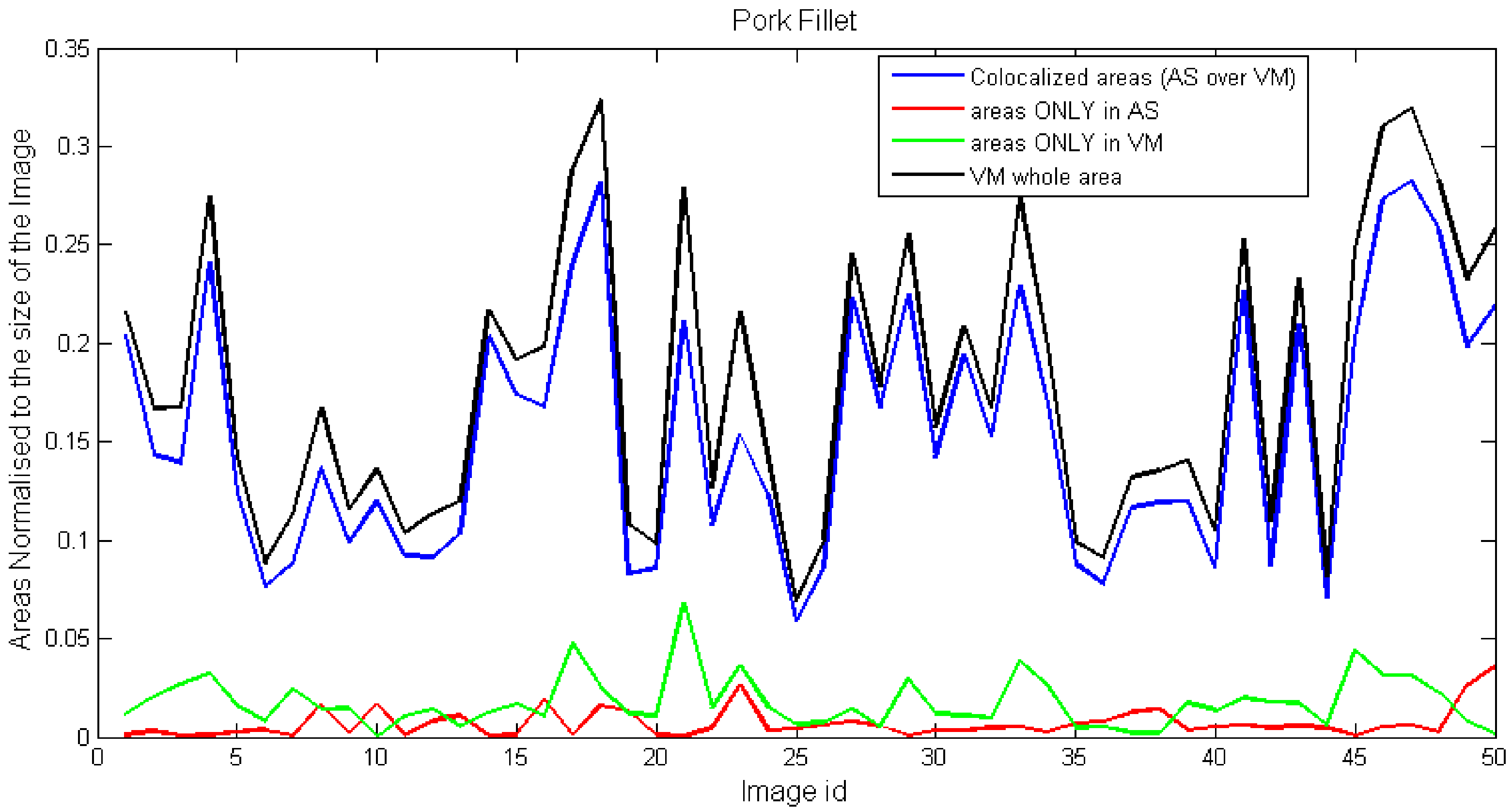


**Figure SI4.5. Pork Fillet.** Areas Normalized to the size of VM area and shown as coverage percentage to the size of the whole image. Black line indicates the informative area detected by VM, blue line the colocalized areas of AS and VM. Green line the area of VM that do not colocalized to AS and red line the AS area not colocalized to VM.

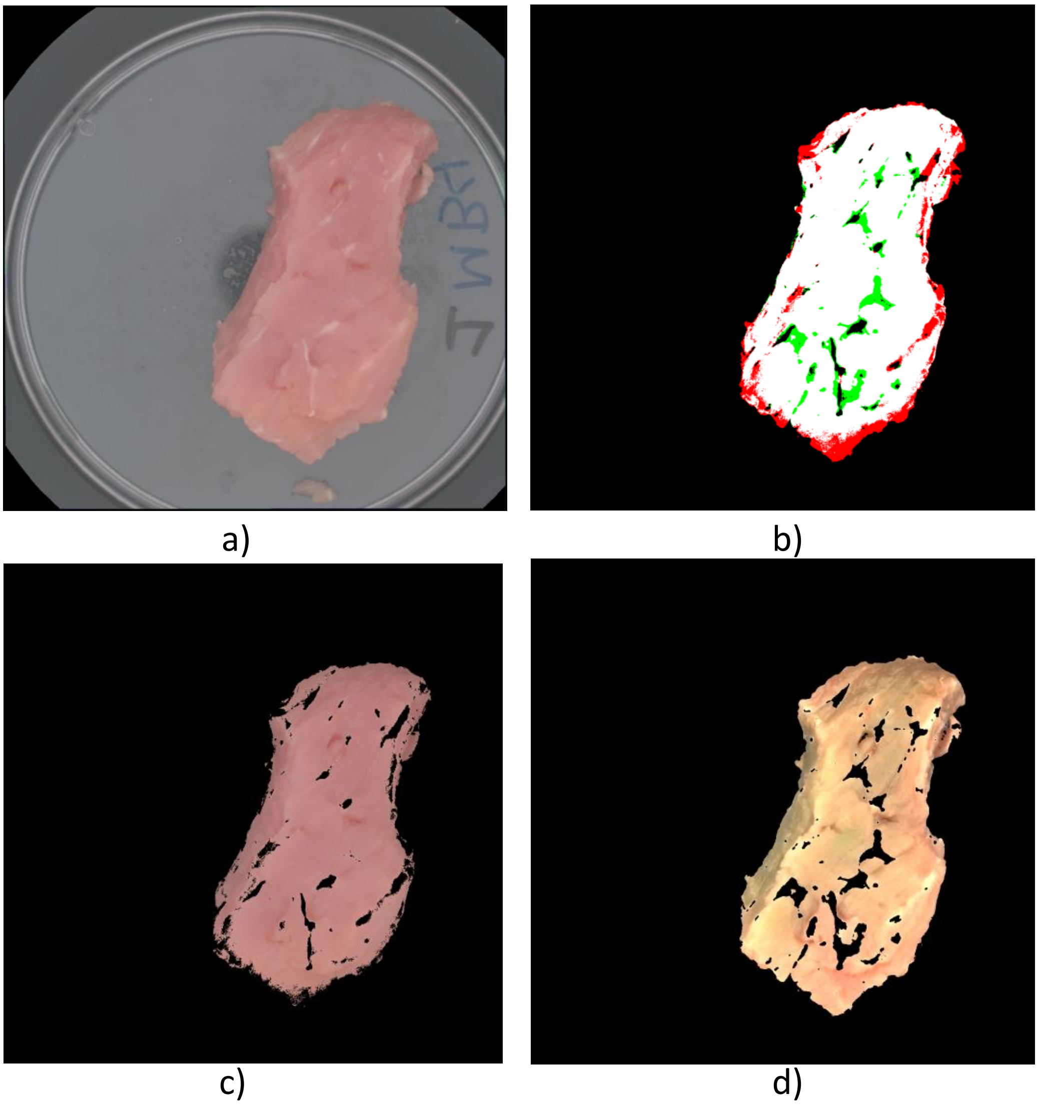


**Figure SI4.6. Pork Fillet.** (a) original pseudo-RGB image, (b) colocalization of AS and VM areas shown in white, green regions indicate the areas detected only by VM, while red areas those that AS have detected, (c) VM informative area, (d) AS informative area.

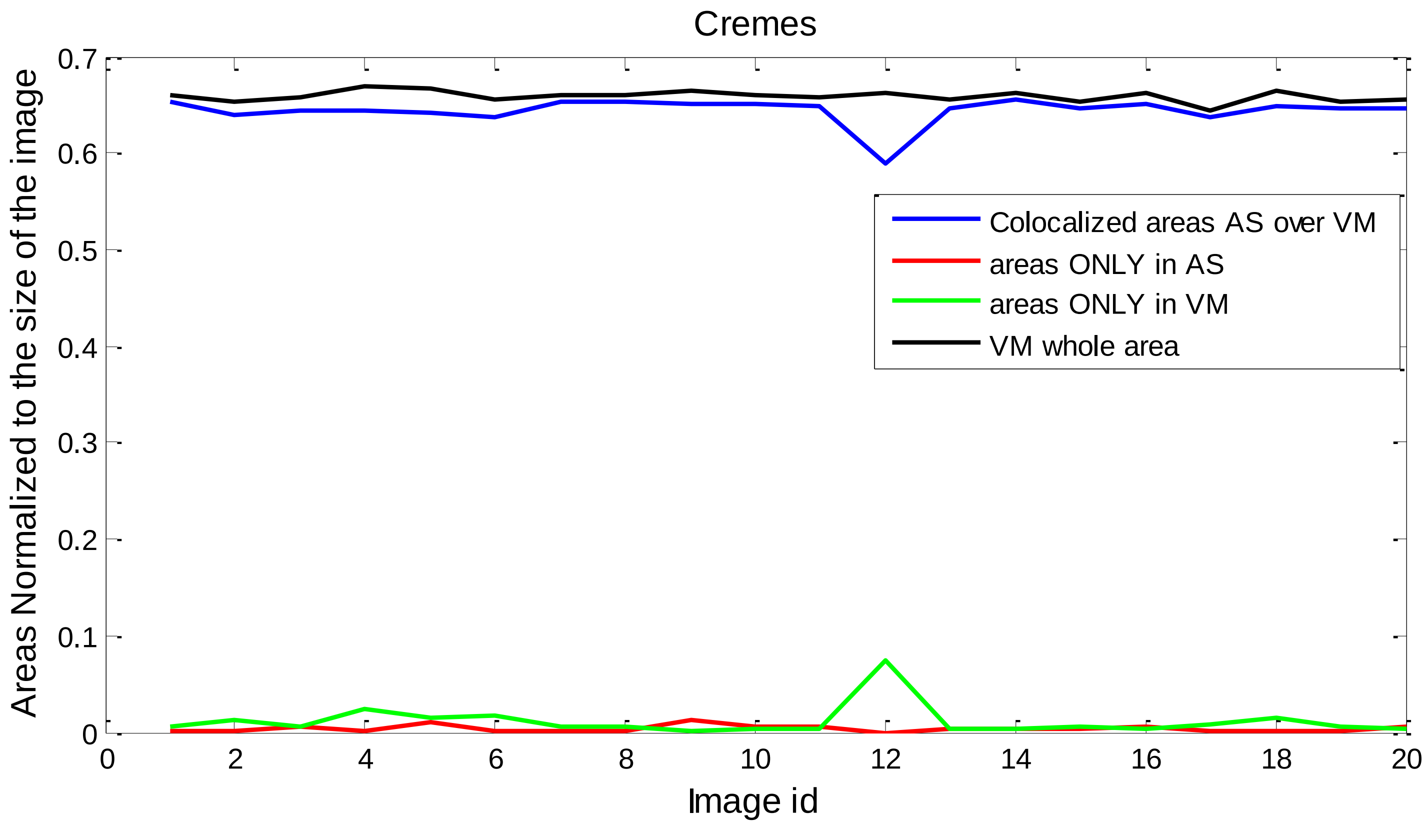


**Figure SI4.7. Cremes.** Areas Normalized to the size of VM area and shown as coverage percentage to the size of the whole image. Black line indicates the informative area detected by VM, blue line the colocalized areas of AS and VM. Green line the area of VM that do not colocalized to AS and red line the AS area not colocalized to VM.

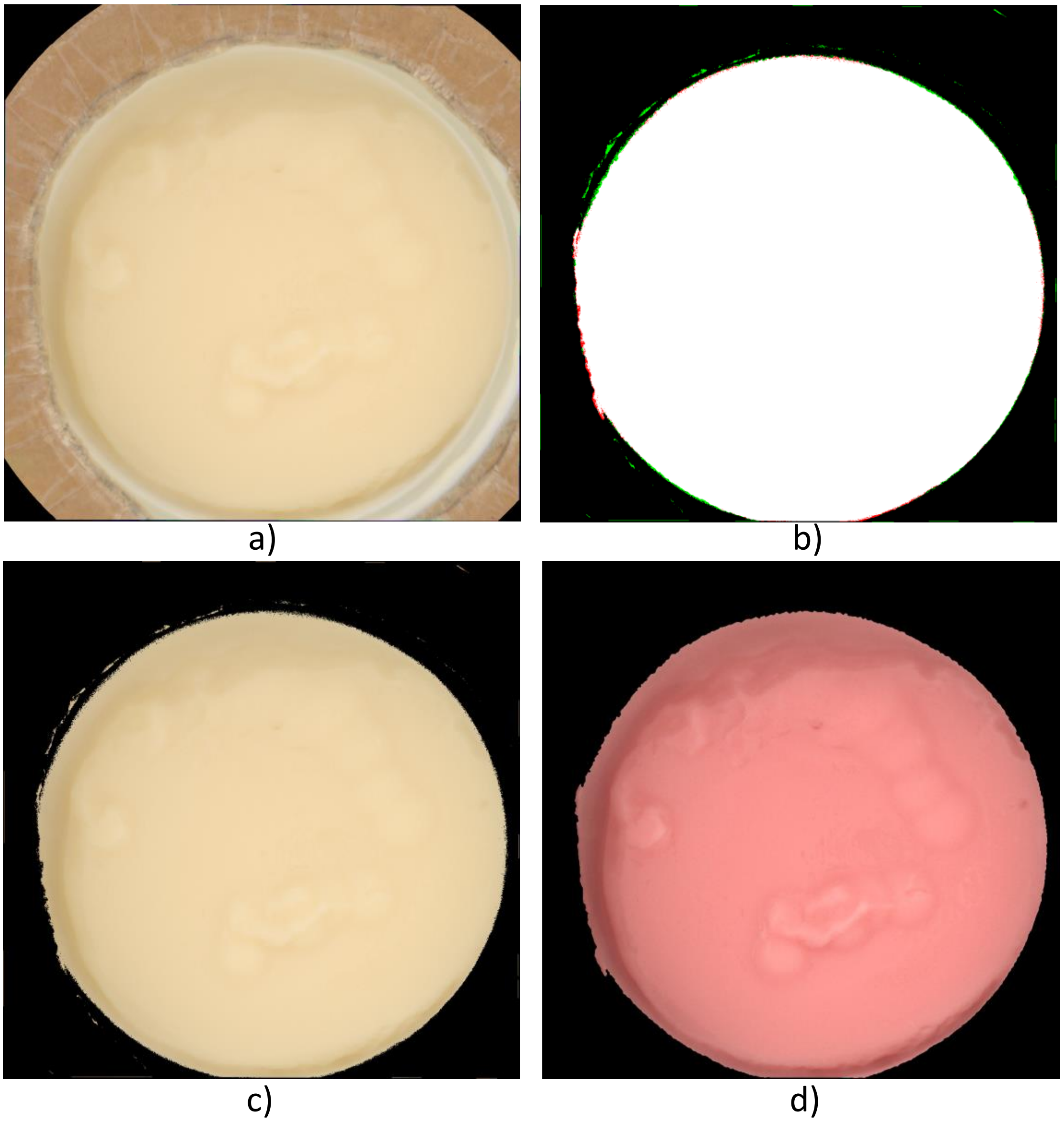


**Figure SI4.8. Crèmes.** (a) original pseudo-RGB image, (b) colocalization of AS and VM areas shown in white, green regions indicate the areas detected only by VM, while red areas those that AS have detected, (c) VM informative area, (d) AS informative area.

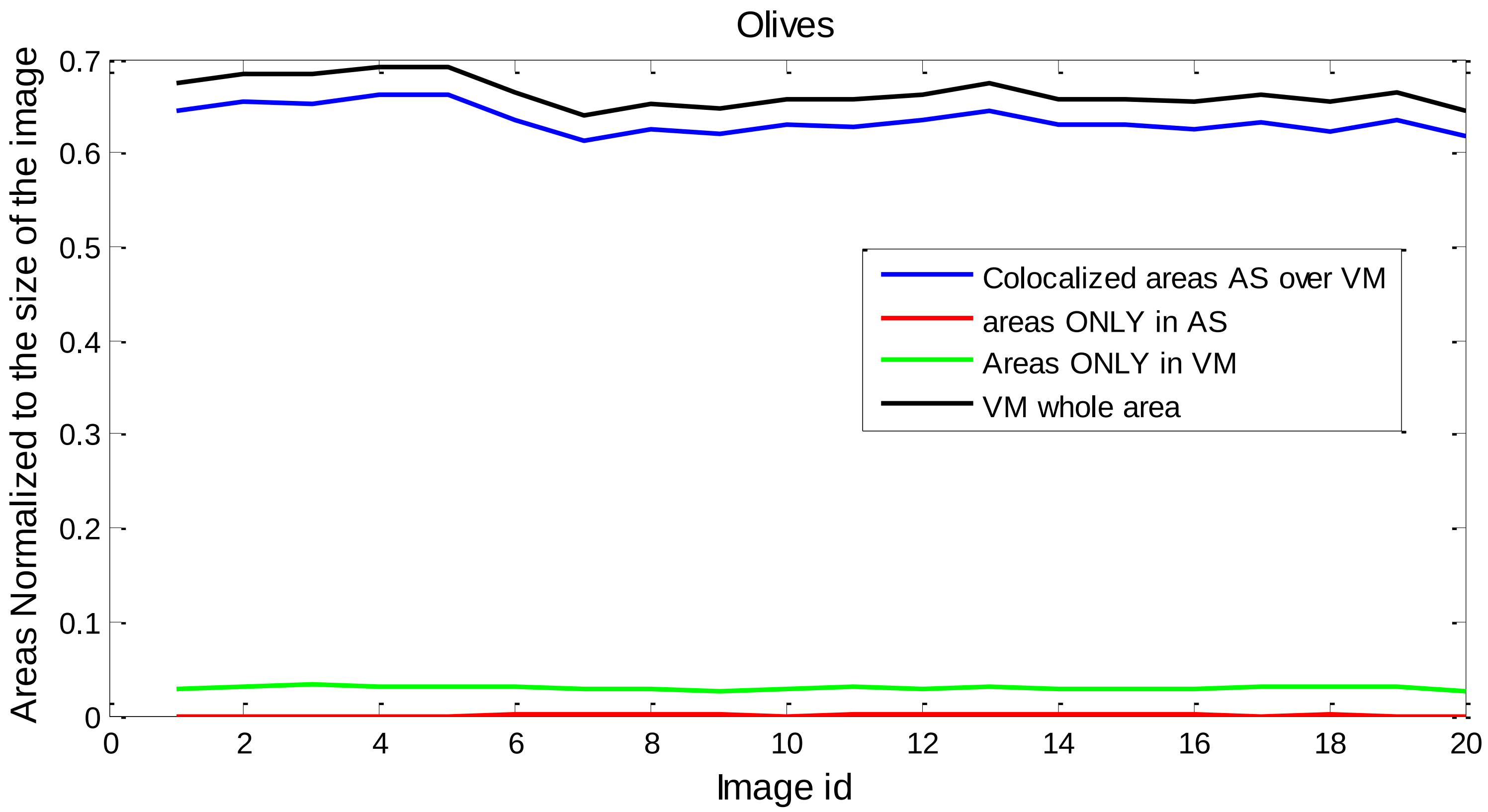


**Figure SI4.9. Olives.** Areas Normalized to the size of VM area and shown as coverage percentage to the size of the whole image. Black line indicates the informative area detected by VM, blue line the colocalized areas of AS and VM. Green line the area of VM that do not colocalized to AS and red line the AS area not colocalized to VM.

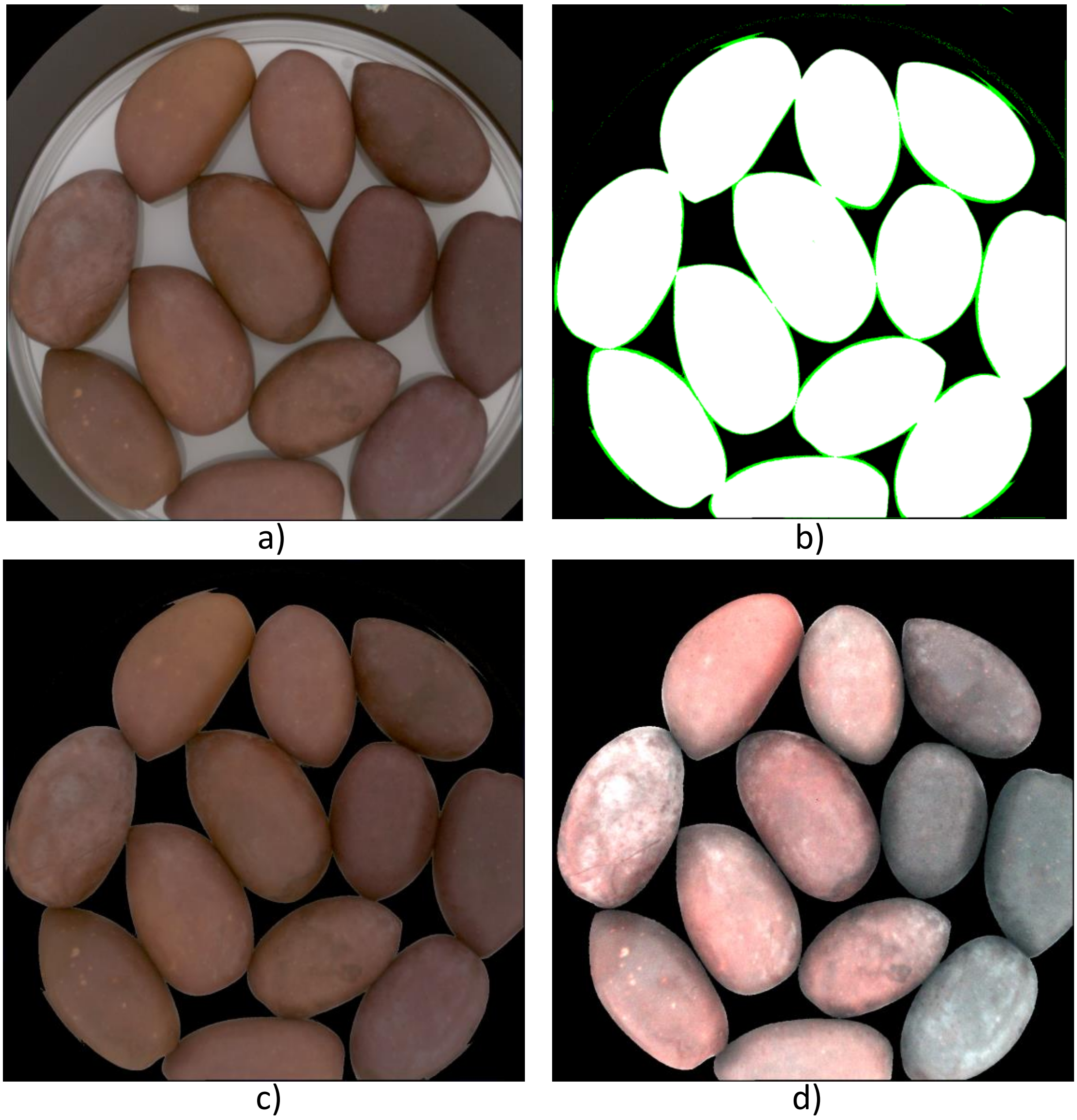


**Figure SI4.10. Olives.** (a) original pseudo-RGB image, (b) colocalization of AS and VM areas shown in white, green regions indicate the areas detected only by VM, while red areas those that AS have detected, (c) VM informative area, (d) AS informative area.
